# The impact of the spatial heterogeneity of resistant cells and fibroblasts on treatment response

**DOI:** 10.1371/journal.pcbi.1009919

**Published:** 2022-03-09

**Authors:** Masud M A, Jae-Young Kim, Cheol-Ho Pan, Eunjung Kim

**Affiliations:** 1 Natural Product Informatics Research Center, Korea Institute of Science and Technology, Gangneung, Republic of Korea; 2 Graduate School of Science and Technology, Chungnam National University, Daejeon, Republic of Korea; Oxford, UNITED KINGDOM

## Abstract

A long-standing practice in the treatment of cancer is that of hitting hard with the maximum tolerated dose to eradicate tumors. This continuous therapy, however, selects for resistant cells, leading to the failure of the treatment. A different type of treatment strategy, adaptive therapy, has recently been shown to have a degree of success in both preclinical xenograft experiments and clinical trials. Adaptive therapy is used to maintain a tumor’s volume by exploiting the competition between drug-sensitive and drug-resistant cells with minimum effective drug doses or timed drug holidays. To further understand the role of competition in the outcomes of adaptive therapy, we developed a 2D on-lattice agent-based model. Our simulations show that the superiority of the adaptive strategy over continuous therapy depends on the local competition shaped by the spatial distribution of resistant cells. Intratumor competition can also be affected by fibroblasts, which produce microenvironmental factors that promote cancer cell growth. To this end, we simulated the impact of different fibroblast distributions on treatment outcomes. As a proof of principle, we focused on five types of distribution of fibroblasts characterized by different locations, shapes, and orientations of the fibroblast region with respect to the resistant cells. Our simulation shows that the spatial architecture of fibroblasts modulates tumor progression in both continuous and adaptive therapy. Finally, as a proof of concept, we simulated the outcomes of adaptive therapy of a virtual patient with four metastatic sites composed of different spatial distributions of fibroblasts and drug-resistant cell populations. Our simulation highlights the importance of undetected metastatic lesions on adaptive therapy outcomes.

## Author summary

Tumors are composed of different cancer cells with varying degrees of treatment resistance, which compete for a shared resource. Adaptive therapy exploits this competition. The paradigm employs patient-specific on and off treatment schedules or lower doses to permit a significant number of drug-sensitive cells to survive. The surviving sensitive cells can suppress the growth of drug-resistant cells via intratumor competition. This competition can be modulated by the spatial structure of tumors. For example, resistant cell configuration, carrying capacity, or migration rate may change local spatial competition between drug-resistant cells or between drug-sensitive and drug resistant cells. In addition, the presence of growth factors produced by stromal cells such as fibroblasts promotes the proliferation of cells, enhancing the competition. To understand the impact of forenamed factors on the outcomes of adaptive therapy, we developed a computational model, 2D on-lattice agent-based model. Our findings show that the spatial factors regulate the local competition and may hold back the benefit of adaptive therapy. Further, the impact of fibroblast depends on the respective positioning of fibroblast to the resistant cells. Finally, we simulated the outcomes of adaptive therapy on multiple metastatic lesions of mixed spatial configuration on a virtual patient. In the simulation, we highlight the importance of undetected metastatic lesions on therapy outcomes.

## Introduction

The current standard of care for the treatment of cancer patients is based on continuous therapy using the maximum tolerated dose (CT-MTD) of cancer drugs with the aim of eradicating drug sensitive cancer cell populations in tumors. Despite the impressive initial tumor responses under CT-MTD, drug resistance inevitably develops in advanced metastatic solid cancers because CT-MTD often selects for drug-resistant cell populations [1, 2]. For example, the majority of patients with metastatic melanomas treated continuously with a BRAF-MEK inhibitor develop resistance over 11–15 months [3, 4]. Drug resistance is known to be a combined consequence of the responses from factors that include intratumor heterogeneity [5, 6], limited drug penetration due to physical barriers [7], and the tumor microenvironment [8–10]. The exploitation of the intratumor competition between heterogeneous cancer cells and the modulation of the tumor microenvironment to bias the selective pressure towards the sensitive cells might have the potential to delay the emergence of resistance.

From an ecological and evolutionary perspective, the net growth rate of a population composed of multiple species is determined by the intrinsic growth rate, death rate, and density-dependent limitations—when multiple species compete for the same resources in a closed environment [11]. This ecological principle implies that the net growth of a tumor cell population can be modulated by inhibiting the intrinsic growth rate of drug-sensitive cells, by increasing sensitive cell deaths, and by modulating the density-dependent growth limitations of drug-resistant cell populations. Because drug resistance often comes with a fitness cost [12, 13], treatment breaks may provide sensitive cells with a higher net growth rate than the resistant cell population. When the intrinsic growth rates of both cell populations are the same, the only way to modulate the growth of resistant cells is to increase density-dependent limitations. Adaptive therapy (AT) is based on this ecological principle of competition between drug sensitive and drug resistant cell population [14]. If kept in a tolerable range, tumor burden is not always lethal [14, 15]. Thus, the objective of AT is to maintain a tolerable tumor burden as long as possible by using treatment holidays or reduced dosing [14]. For example, under AT, a patient is treated with therapy from the diagnosis until the tumor burden falls to a fraction of the initial cell population (e.g., 50% of the initial burden [16]). The goal is to reduce the cell population to an acceptable level that has sufficient sensitive cells to maintain density-dependent competitive stress on the growth of resistant cells. Then, a treatment break is scheduled to allow the remaining sensitive cells to grow and to limit the growth of the resistant cell population by leveraging competition. Once the total cell population is back to the initial level, a treatment is administered again. This on–off treatment cycle is repeated until the tumor progresses. This adaptive therapy strategy has been shown to have some degree of success in both preclinical experiments [17, 18] and a clinical trial [16]. In particular, a clinical trial for prostate cancer therapy showed that adaptive therapy can delay disease progression for 27 months by using only a 53% cumulative drug rate compared to CT-MTD [16].

Several mathematical and computational models have been developed to compare AT with CT-MTD in various scenarios, utilizing two quantification metric, time to relapse and tumor progression (TTP) and time gain (TG). TTP is the time at which the tumor progresses. The time gain (TG) quantifies how many days gained by AT from CT-MTD (TG = TTP in AT—TTP in CT-MTD). Gallaher et al. developed an off-lattice agent-based model to simulate the impact of cancer cell heterogeneity and space on AT outcomes. They reported an extension of TTP of about one year under AT compared to CT-MTD (CT-MTD: 400 days vs. 700 days) [19]. Gatenby et al. developed a model consisting of five types of cells with differential drug responses and showed that tumor cells under CT-MTD grow to a carrying capacity by about 2400 days, while under AT, the tumor burden was kept under control at 20% of the carrying capacity [14]. A mathematical model in [16] showed that the on–off cycling rate of treatments depends on cell–cell competition and initial tumor cell population composition [16], where the threshold for treatment breaks was 50% of the initial tumor burden. A different threshold for treatment breaks was considered by Hansen and Read [20, 21]. This study further demonstrated that a 20% reduction threshold resulted in more delayed progression than a 50% reduction for different degrees of initial resistance [20, 21]. Kim et al. identified predictive factors that determine the benefit of AT. In the case of melanoma, the initial tumor burden, growth rate, switching rate, and competition coefficient were identified as crucial parameters for deciding the TG of AT by using CT-MTD [21]. The initial proportion of resistance is another contributing factor. Strobl et al. showed that an 1% initial resistance delayed the progression by up to 211 days for an initial burden of 75%, while 10% resulted in almost no TG [22]. A game-theoretical model was used to propose a combination of strategy for AT [23, 24]. Recently, Viossat and Noble [25] provided theoretical conditions for the maximization of the benefits of AT. In particular, they provided an explicit formula for TG under AT, which included the intensity of competition between drug-sensitive and drug-resistant cells, the most critical factor. A couple of spatial models have investigated the consequences of spatial heterogeneity on tumor growth and treatment outcomes. Agent-based models have shown that even lower doses can limit tumor growth if resistant cells are spatially restricted by sensitive cells [19, 26]. Tumors with randomly spread resistant cells were reported to grow much faster than tumors with resistant cells that were clustered together [26, 27].

Tumor microenvironment can modulate tumor progression and treatment outcomes. For example, cancer associated fibroblasts (CAFs), a group of activated fibroblasts in tumors, are known to promote individual cancer cells’ growth and migration by producing growth factors and an extracellular matrix [28–33]. Recent experimental studies have reported the impact of spatial location of CAFs on treatment outcomes. Marusyk et al. demonstrated that physical proximity to CAFs determines tumor cell survival under therapy [30]. Tumor cells that are close to CAFs can survive longer under therapy due to the fibroblast-mediated elevation of the threshold of drug concentration required for cell death and the lower rate of drug activity due to the physical barrier against drug penetration (i.e., collagen) generated by fibroblasts. The analysis of the geospatial distribution of cancer-associated fibroblasts in metastatic clear cell renal cell carcinoma suggested that proximity clustering of tumor cells with the fibroblasts resulted in worse overall survival and resistance to targeted therapy [34].

The impact of CAFs, in particular its spatial distribution, on AT outcomes has yet to be investigated. Here, we developed a 2D on-lattice agent-based model (ABM) to simulate the effects of the different distribution of CAFs and resistant cell populations on the therapy outcomes. Specifically, we considered three different initial cell configurations of resistant cell populations, namely, clumped, random, and uniform. The spatial distribution of CAFs appears to be diverse. For example, in the ovarian tumor, clumps of tumor cells surrounded by fibroblasts were observed (Fig 1 in [35]). Also, side-wise alignment of fibroblasts to tumor cells was observed in ovarian cancer (Fig 2 in [35], Fig 1 in [36]). A random scattering of fibroblasts throughout the tumor was observed in prostate cancer(Fig 3 in [32]). Random scattering of clumps of fibroblast was found in lung cancer (Fig 2 in [37]) as well. For simplicity, we considered five different spatial arrangements of CAFs, which may represent a fibroblast distribution in a tumor. In addition, we simulated the outcomes of AT on a virtual patient with four metastatic sites composed of different spatial distributions of fibroblasts and resistant cell populations.

## Materials and methods

We developed a 2D on-lattice agent-based model, representing a small primary tumor or a metastatic lesion. For simplicity, we assume that a tumor cell population can be classified into two types of cells: drug-sensitive (S-cell) and drug-resistant (R-cell). We denote the total cell population, S-cell population, and R-cell population at time *t* with *N*(*t*), *S*(*t*), and *R*(*t*), where *N*(*t*) = *S*(*t*) + *R*(*t*).

### Initial and boundary conditions

**Fig 1:**
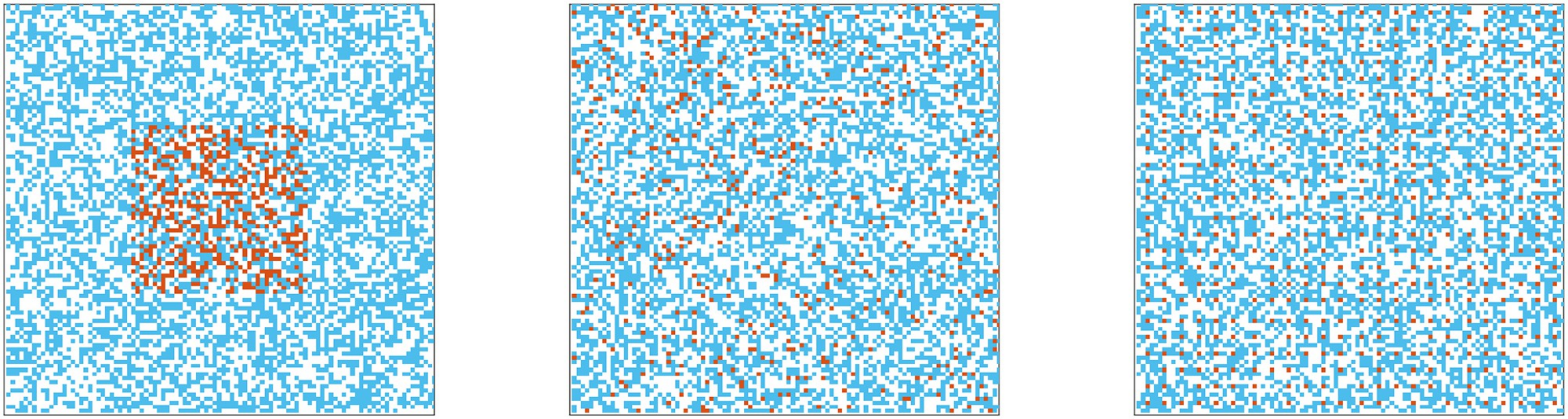
Initial cell configurations. Three types of initial cell configurations. Orange dots: R-cells, blue dots: S-cells, white dots: empty sites. Clumped R cells (first), random R-cells (center), and uniform R-cells (right).

We assume that a percentage *f*_0_ of the initial cells (*N*(0)) is resistant (i.e., $R\left(0\right)=\frac{f_{0}}{100}N\left(0\right)$ and $S\left(0\right)=(1-\frac{f_{0}}{100})N\left(0\right))$. Initially, a total of *S*(0) cells is randomly dispersed over the domain, while a total of *R*(0) cells is placed in three different dispersion patterns—random (*r*), uniform (*u*), or clumped (*c*)—in the domain Fig 1 [38]. In the clumped case, all of the R-cells were randomly dispersed in a square centered in the middle of the domain, where the same number of R-cells were randomly dispersed over the whole domain in the random case. On the other hand, in the uniform case, all of the R-cells were manually placed to maximize the distance between R-cells over the whole domain. The boundary is assumed to be closed (no cell leaving the boundaries).

**Fig 2:**
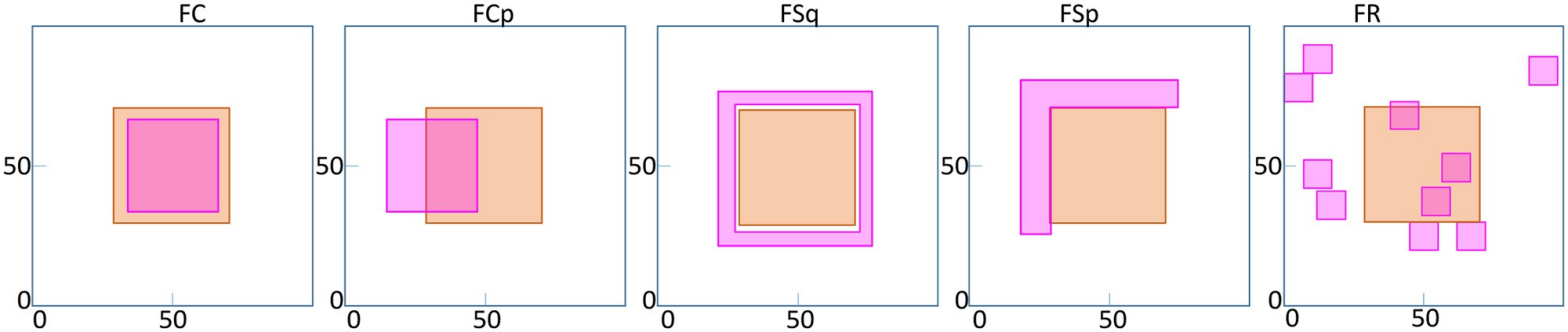
Fibroblast distribution. The pink shaded regions show the five types of representative CAF distribution (FC, FCp, FSq, FSp and FR). The orange square at the centre is the clump of R-cells. The dimensions have been provided in the text.

Spatial allocation and the mechanism of modulating tumor growth by CAFs is a complex and multifaceted phenomenon [31, 39]. To gain a theoretical understanding while keeping the problem simple, we consider the following five different CAF configurations (Fig 2).

- NoF: No fibroblast.
- FC: Fibroblast over a 32 × 32 square overlapping with the R-cell clump at the center.
- FCp: Fibroblast partially overlapping with the R-cell clump at the center, diagonally located between the sites (17, 35) and (49, 66).
- FSq: Fibroblast over a hollow square with an outer dimension of 55 and inner dimension of 45, diagonally situated between the sites (23, 23) and (77, 77), with a wall thickness of five lattices. In this case the fibroblast completely surrounds the R-cell clump.
- FSp: Fibroblast over a “L” shaped region with vertices (21, 25), (30, 25),(30, 71),(75, 71),(75, 80) and (21, 80). In this case, the fibroblast partially surrounds the R-cell clump.
- FR: 10 Fibroblast clumps of size 10 × 10 randomly placed in the domain.

In all the cases defined above, we assumed that CAF comprises of about 10% of the domain. Fibroblasts are observed to promote cancer cell growth up to about 5 times [32, 33] We assume fibroblast-mediated cell proliferation rate *r*_*iF*_ = *αr*_*i*_, where *i* ∈ {*S*, *R*}, and simulate our model for *α* = 2, 4 [32, 33]. In this study, we assume that cancer cells in the fibroblast location have CAF-mediated growth promotion for simplicity.

### Cell-cycle decision

**Fig 3:**
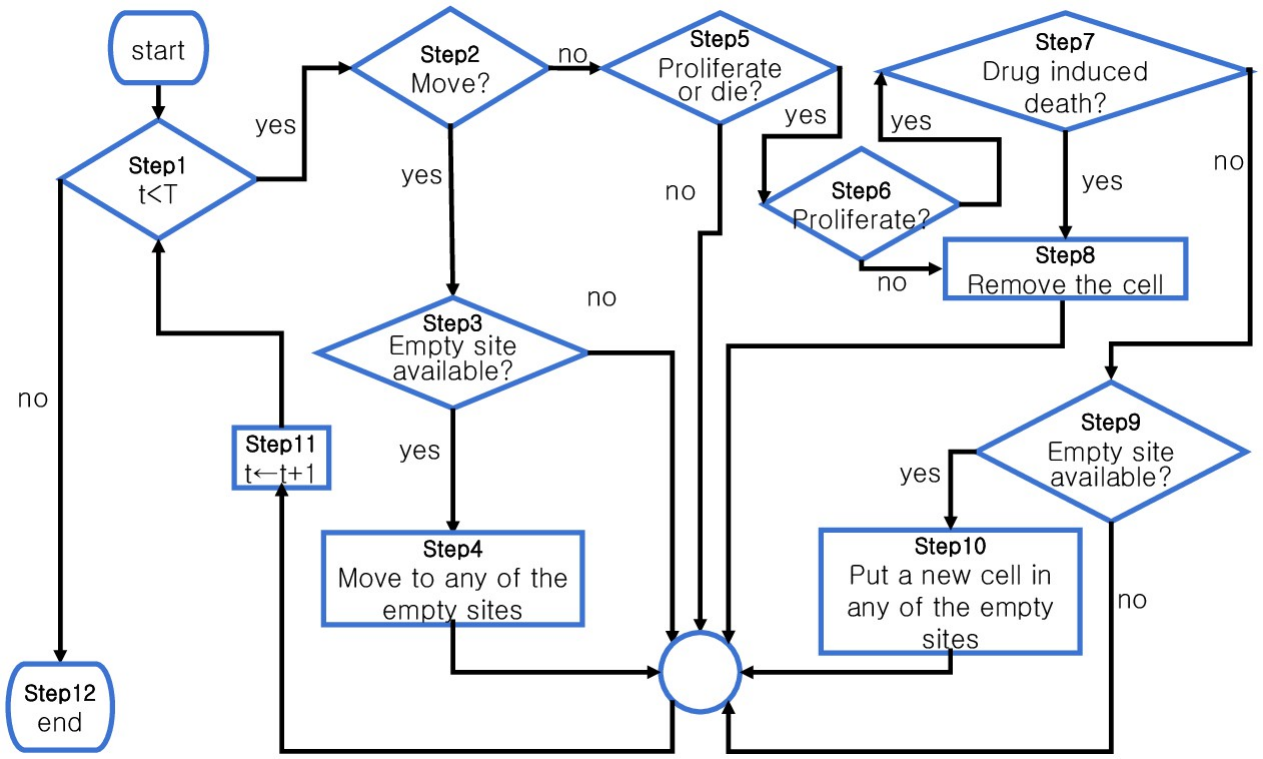
Cells’ life cycle. Flow chart of the cells’ life cycle. In each time step, all of the cells follow the steps in the flow chart.

Each cell occupies a lattice point in a square domain of size *l* × *l*. In every time step, each cell may stay stationary, or it can move, divide, or die. The S-cells and R-cells divide at a constant rate of *r*_*S*_ or *r*_*R*_, respectively. In this study, we considered the von Neumann neighborhood (VNHD), which is composed of the sites on the east, west, south, and north of each cell. The death rate of both types of cells is *d*_*T*_. The drug concentration *D*(*t*) is homogeneous in the domain, and a drug-induced death rate (*δ*_*D*_) is applicable to S-cells only. A sensitive cell undergoing mitosis can be killed by a drug with a probability of *δ*_*D*_*D*(*t*). Both S-cells and R-cells follow the rules described in the flow chart in Fig 3. A brief explanation of the flow chart (Fig 3) is provided in the following.

- Step 1: If *t* < *T*, where *T* is the end time, go to Step 2; otherwise, go to Step 12.
- Step 2: Decide whether the cell will move. Pick a random number from a uniform distribution (*x*_*m*_ ∼ U[0, 1]). If *x*_*m*_ < *mr*_*S*_, where *mr*_*S*_ is the probability of cell migration, then go to Step 3. If not, go to Step 5.
- Step 3: Is one of its VNHDs empty? If yes, go to Step 4. If not, go to Step 11.
- Step 4: Randomly move the cell to one of the empty sites in the VNHD. Go to Step 11.
- Step 5: Decide whether the cell will divide or die. Pick a random number from a uniform distribution (*x*_*pd*_ ∼ U[0, 1]). If *x*_*pd*_ < *r*_*j*_ + *d*_*T*_ with *j* ∈ {*S*, *R*}, where *r*_*j*_ is the j-cell proliferation rate and *d*_*T*_ is the normal cell death rate, then go to Step 6. If not, go to Step 11.
- Step 6: Decide whether the cell will divide. Pick a random number from a uniform distribution (*x*_*p*_ ∼ U[0, 1]). If $x_{p}<\frac{r_{j}}{r_{j}+d_{T}}$, then go to Step 7. If not, go to Step 8.
- Step 7: Decide whether the cell will die due to the drug. If (*x*_*d*_ ∼ U[0, 1]) and if *x* < *δ*_*D*_*D*(*t*), where *δ*_*D*_ is the probability of cell death (for R-cells, *δ*_*D*_ = 0), go to Step 8. If not, go to Step 9.
- Step 8: Remove the cell, make the site empty, and go to Step 11.
- Step 9: Is one of its VNHDs empty? If yes, go to Step 10. If not, go to Step 11.
- Step 10: Randomly put a new cell of the same type in VNHD. Go to Step 11.
- Step11: *t* ← *t* + 1. Go to Step 1.
- Step12: The simulation ends.

### Number of cells in the neighborhood

To quantify local cell–cell competition, we introduce the following notation.

$$\begin{array}{c} N_{jk}^{i}\left(t\right)=\text{number}\;\text{of}\;j\text{-cells}\;\text{around}\;\text{a}\;k\text{-cell}\;\text{at}\;\text{time}\;t\;\text{with}\;i\;\text{initial}\;\text{cell}\;\text{configuration} \end{array}$$

where, *j* ∈ {*R*(R-cells), *S*(S-cells), *E*(Empty site)}, *k* ∈ {*R*, *S*} and *i* ∈ {*c*(Clumped), *r*(Random), *u*(Uniform)}. To denote the mean over all of the *k*-cells in the domain at time *t*, we write $N_{j\bar{k}}^{i}\left(t\right)$.

We denote the number of empty sites by $N_{Ek}^{i}$ ($0\leq N_{Ek}^{i}\leq 4$). A cell can move to any unoccupied sites in its VNHD provided that $1\leq N_{Ek}^{i}\leq 4$. During cell proliferation, one parent cell divides into two daughter cells of the same type. To accommodate the daughter cell, at least one empty site is required ($1\leq N_{Ek}^{i}\leq 4$) in the parent cell’s von Neumann neighborhood. If $N_{Ek}^{i}=0$, the proliferation was not executed. Upon cell division, one daughter cell is placed in the parent cell’s location, and the other is randomly placed in one of the empty sites in the VNHD. Upon the availability of an empty site in the VNHD (i.e., $1\leq N_{Ek}^{i}\leq 4$), while attempting to divide, the mother S-cell may die with a probability of *d*_*D*_ due to the drug, but the R-cells do not experience drug-induced death. Dead cells are immediately removed from the respective sites.

### Model parameters

As a representative structure, we assume a square domain of 100 × 100 lattice points. We simulate our model for *N*(0) = 5000, 7000 and *f*_0_ = 10%, 1%. The S-cells are assumed to be randomly dispersed over the domain. In the clumped case, all of the R-cells are randomly dispersed in a 40 × 40 clump. The parameters employed in this study are summarized in Table 1.

**Table 1:** The parameter values are listed in the following table.

Parameter	Description	Value	Reference
*N*(0)	Initial number of cells	5000, 7000	Assumed
*K*	Carrying capacity of each lattice point	1, 2	Assumed
*f*_0_	Initial percentage of the resistant cell population (*R*(0)/*N*(0))	1%, 10%	[40]
*r*_*S*_	Sensitive cell proliferation rate	0.027 per day	[16]
*r*_*R*_	Resistant cell proliferation rate	(1–0.3)*r*_*S*_	[19]
*d*_*T*_	Cell death rate	0.3*r*_*S*_	[22]
*m*	Migration rate	[0, 4*r*_*S*_]	Assumed
*D*(*t*)	Drug concentration at time *t*	0, 1	
*δ*_*D*_	Drug-induced death rate of S-cells	0.75	[23]
*ρ*	AT threshold for treatment break	0.5	[16]
*r*_*SF*_	Fibroblast-mediated sensitive cell proliferation rate	2*r*_*S*_, 4*r*_*S*_	Assumed
*r*_*RF*_	Fibroblast-mediated resistant cell proliferation rate	2*r*_*R*_, 4*r*_*R*_	Assumed

### Treatment schedules

We consider two treatment strategies: continuous administration of maximum tolerated dose (CT-MTD) and adaptive therapy (AT). In a CT-MTD therapy, the maximum tolerated dose is applied to the domain over the entire simulation time. In an AT simulation, the treatment is provided from the beginning of the simulation until the cell population is reduced to *ρN*(0). The treatment is stopped until the total population, *N*(*t*), reaches *N*(0) again. Then, the treatment is re-applied. In this study, we assume that the MTD is applied during the treatment cycle and that *ρ* = 0.5 [16]. In mathematical notation, drug concentration can be written as follows. We consider the time when the total population reaches 120% of *N*(0) as the time to tumor progression (TTP).

$$\begin{array}{c} CT-MTD:D(t)=\text{MTD}\;\text{for}\;t\geq 0 \end{array}$$

$$\begin{array}{c} AT:D\left(t\right)=\{\begin{array}{cc} \text{maximum}\;\text{tolerated}\;\text{dose} & \;\text{until}\;N(t)<\rho N(0),\rho =0.5,\\ 0 & \;\text{until}\;N(t)\geq N(0) \end{array} \end{array}$$

### Simulation

The model was implemented on the JAVA platform using the Hybrid Automata Library (HAL) [41]. The generation of the initial cell configuration, the data analysis, and the visualization were performed by using MATLAB. To keep the results unbiased, the sequence of cells in the simulation was shuffled at the beginning of every time step. For each simulation scenario, we simulated 30 virtual tumors (i.e., 30 realizations of the model simulation), unless otherwise noted. To denote the average over the 30 simulations, we used over-bars, such as $\bar{N}\left(t\right)$ and ${\bar{N}}_{jk}^{i}\left(t\right)$.

### Statistical analysis

To investigate the consequences of a parameter change in the results of the 30 realizations, we used a two-sample t-test. Significant differences with p-value < 0.001, 0.01, and 0.05 are represented by triple asterisk(***), double asterisk(**), and asterisk(*), respectively. For non-significant differences, we use “n.s.”.

## Results

### Impact of the initial R-cell configuration on the TTP under CT-MTD

**Fig 4:**
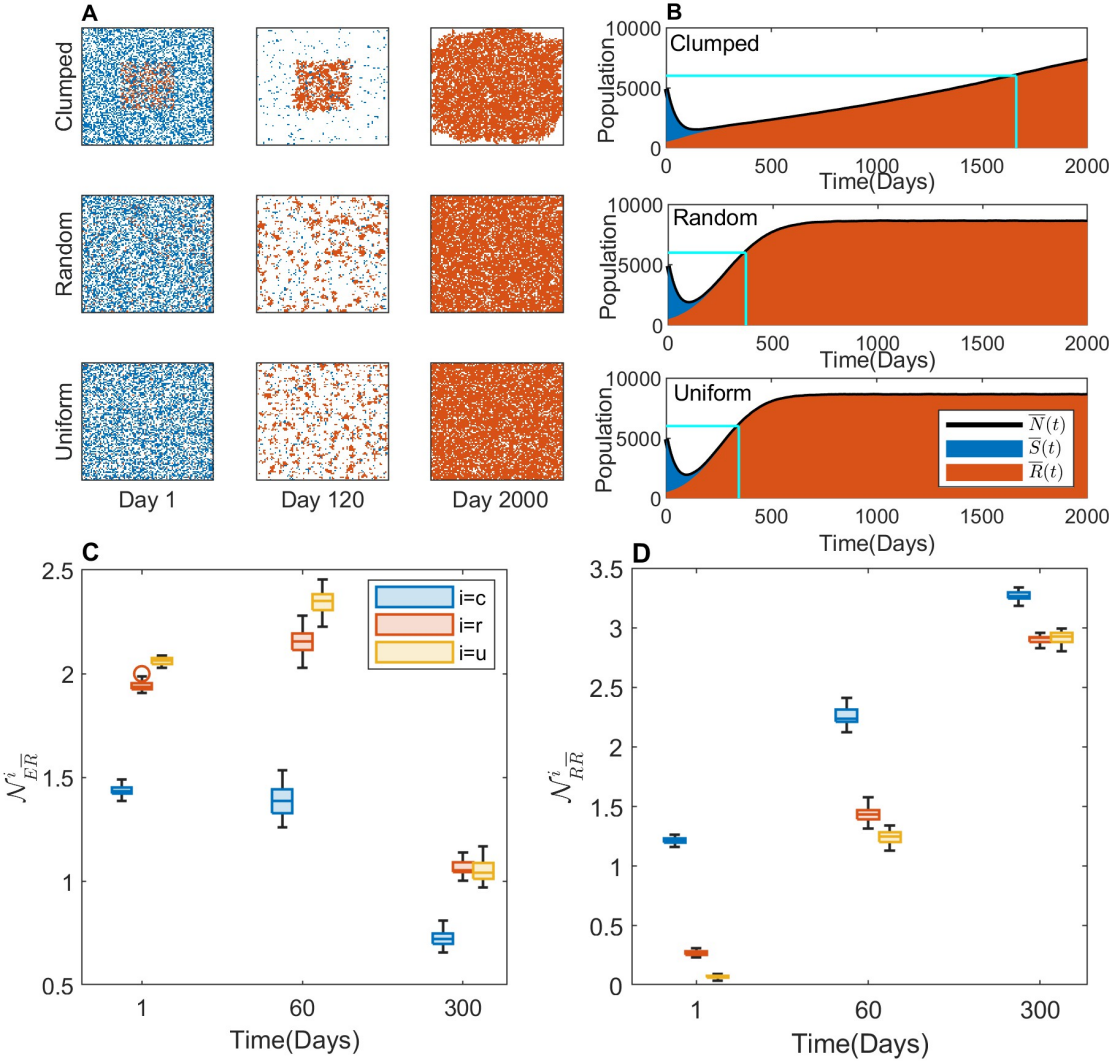
Effects of initial R-cell distribution on the TTP under CT-MTD. (**A**) The cell configurations on days 1, 120, and 2000 are shown. The blue, orange, and white dots are S-cells, R-cells, and empty sites, respectively. (**B**) The average temporal evolution of the average number of S-cell and R-cell populations over 30 realizations with clumped (upper panel), random (middle panel), and uniform (bottom panel) initial cell configurations (blue: S-cell, orange: R-cell). Black solid line: average total cell population ($\bar{N}\left(t\right)=\bar{S}\left(t\right)+\bar{R}\left(t\right)$). Vertical cyan line: TTP in each case; horizontal cyan line: the 120% level of the initial tumor volume (tumor progression threshold). The average numbers of empty sites ($N_{E\bar{R}}^{i}$) and R-cells ($N_{R\bar{R}}^{i}$) in the VNHD of an R-cell in the 30 realizations are shown as boxplots in (**C**) and (**D**), respectively, for *i* = *c*, *r*, *u*. The blue, orange, and yellow boxes are for the clumped (*c*), random (*r*), and uniform (*u*) cases, respectively.

First, we simulated CT-MTD on the three types of initial cell configurations for a time span of 2000 days. In this simulation, we assumed the carrying capacity of each lattice point to be *K* = 1 and the cell migration rate to be *m* = 0. Under the therapy, the S-cells died out quickly, and the remaining R-cells started to grow and fill the model domain. The representative spatial distributions of the tumor cells are shown in Fig 4A on the 1^*st*^, 120^*th*^, and 2000^*th*^ day. The cell configuration in the clumped case was significantly different from those in the random and uniform cases, between which the difference seemed to be negligible. On the 120^*th*^ day, slightly larger patches of resistant cells are observed in the random case than in the uniform case. By the end of the simulation, the whole domain was captured by R-cells in both cases. On the other hand, in the clumped case, the R-cells grew in a patch in the center. By the end of the 2000 days, a huge clump of R-cells captured almost the entire domain (S1 Movie).

The temporal dynamics of different types of cells are presented in Fig 4B. The TTP (time to progression) in the three cases were 1662 day, 372 day, and 345 day, respectively. The dynamics of the S-cells were almost the same for all three types of initial configurations (S1A Fig), as they were initially similarly sparse and had the same growth parameters in all cases. The growth dynamics of R-cells in clumped case is different from both random and uniform cases, resulting in different TTP. To examine the reason for why the TTP was significantly different in the clumped case compared to the random and uniform cases, we investigated the local R–S and R–R spatial competition. Specifically, we calculated the numbers of S cells, R cells, and empty sites of each R-cell VNHD in the three spatial patterns.

To compare the local growth potential of R-cells in the three spatial patterns, we calculated the number of empty sites in the VNHD of each R-cell. The average number of empty sites in the neighborhood of an R-cell was lower in the clumped case than in both the random and uniform cases ($N_{E\bar{R}}^{c}<N_{E\bar{R}}^{r},N_{E\bar{R}}^{u}$). Fig 4C shows that the number of empty sites around each R-cell increased from day 1 to day 60 in the random and uniform cases ($N_{E\bar{R}}^{r}$ and $N_{E\bar{R}}^{u}$ increased from day 1 to 60) because the treatment-induced deaths of S-cells freed up space in the neighborhood of each R-cell, leading to a reduction in R–S spatial competition.

We next compared the R–R local competition by quantifying the average number of R-cells in the VNHD of each R-cell. During CT-MTD, the number of R-cells in the neighborhood of each R-cell increased over time in all three cases due to growth of R-cells. In the clumped case, the R-R competition was significantly higher than the competition in the random or uniform case ($N_{R\bar{R}}^{c}>N_{R\bar{R}}^{r},N_{R\bar{R}}^{u}$) (Fig 4D: blue boxes vs. yellow and orange boxes), resulting in a delayed TTP.

### Impact of the initial R-cell configuration on the TTP under AT

**Fig 5:**
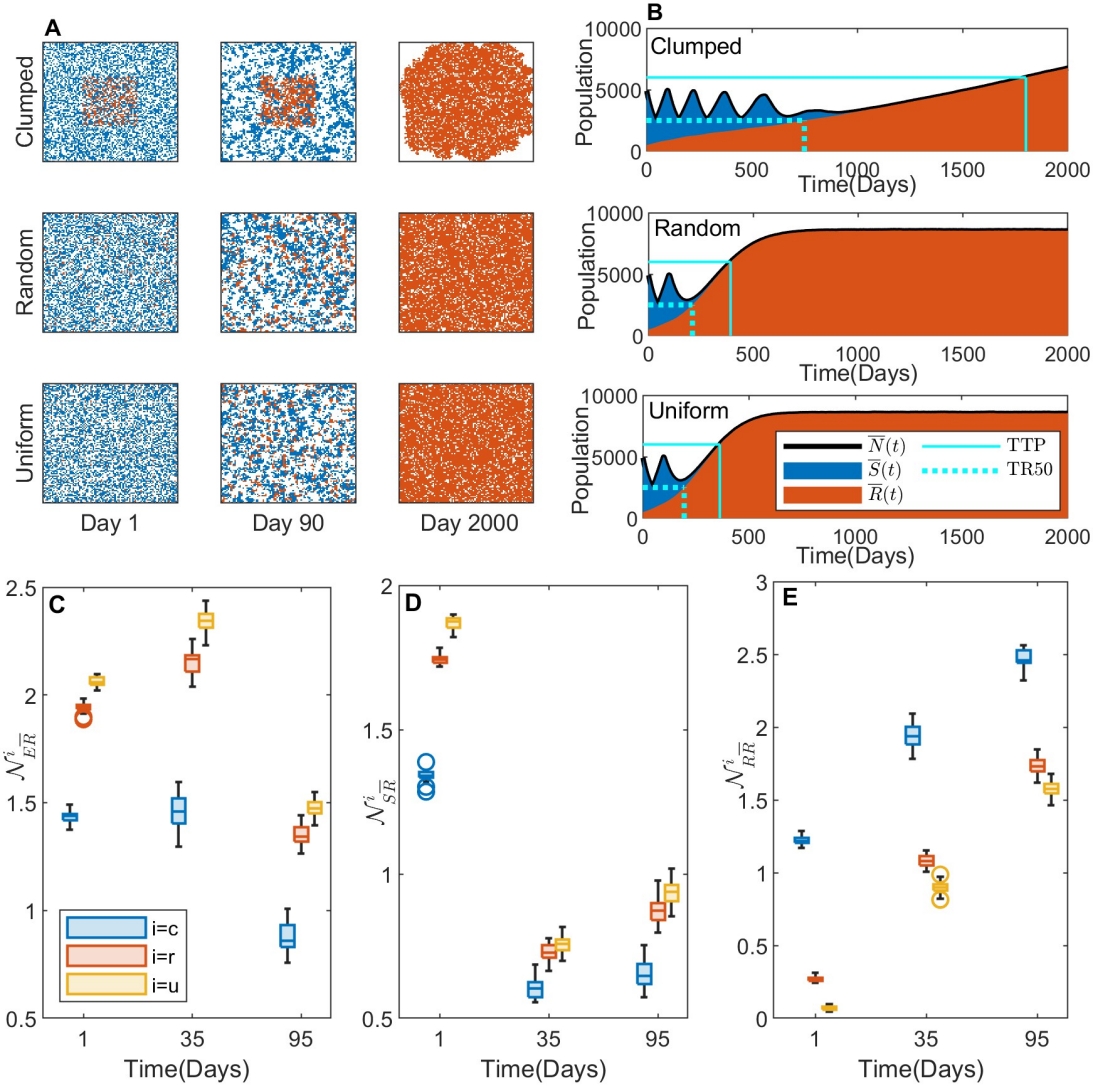
Effect of the initial R-cell distribution on the TTP under AT. (**A**) Cell configurations on days 1, 120, and 2000. The square signifies the domain representative of the tumor. The blue, orange, and white dots are S-cells, R-cells, and empty sites, respectively. (**B**) Average temporal evolution of the S-cell and R-cell populations for the clumped (upper panel), random (middle panel), and uniform (bottom panel) cases of the initial cell configurations (blue dots: S-cells, orange dots: R-cells). Black solid line: the total population (*N*(*t*) = *S*(*t*) + *R*(*t*)). Vertical solid cyan line: TTP; horizontal solid cyan line: 120% level of the initial tumor volume. Vertical dotted cyan line: time for the R-cells to reach 50% of the initial tumor volume (TR50); horizontal dotted cyan line: 50% level of the initial tumor volume. The average numbers of empty sites ($N_{E\bar{R}}^{i}$), S-cells ($N_{S\bar{R}}^{i}$), and R-cells ($N_{R\bar{R}}^{i}$) in the VNHD of an R-cell in the 30 realizations are shown as boxplots in (**C**), (**D**), and (**E**), respectively, for *i* = *c*, *r*, *u*. The blue, red, and yellow boxes are for the clumped (*c*), random (*r*), and uniform (*u*) cases, respectively.

Next, we investigated the effect of the initial R-cell distribution on the AT outcomes. Fig 5A and S1 Movie shows a representative cell configuration at different times for AT with three different initial R-cell distributions. The cell population growth presented in Fig 5B shows that the TTP values were 1776, 392, and 362 days in the clumped, random, and uniform cases, respectively. The total cell population went through four on–off treatment cycles until the TTP in the clumped case. In the other two cases, only one on–off treatment cycle was allowed until the TTP. To understand the mechanism by which AT caused a more delayed TTP in the clumped case compared to the random or uniform case, we first investigated the local growth potential on days 1, 35, and 95. We chose the 35th day (when the first cycle had yet to finish) and the 95th day (after which the second cycle started) for all the cases. The average numbers of empty sites (Fig 5C) and S-cells (Fig 5D) in each R-cell neighborhood were lower in the clumped case than in the other two cases ($N_{E\bar{R}}{\left(t\right)}^{c}<N_{E\bar{R}}^{r}\left(t\right)$,$N_{E\bar{R}}^{u}\left(t\right)$ (Fig 5C). The number of empty sites in the neighborhood of a each R-cell ($N_{E\bar{R}}^{c}$) did not significantly change from day 1 to day 35, although the numbers in both the random and uniform cases ($N_{E\bar{R}}^{r}$ and $N_{E\bar{R}}^{u}$) increased remarkably. During the first treatment break (from day 35 to day 95), the S-cells divided, filling up empty sites in the neighborhoods. This resulted in a reduction of $N_{E\bar{R}}{\left(t\right)}^{c,r,u}$ (Fig 5C: boxplots on day 95 vs. boxplots on day 35).

Next, we compared the intensity of the spatial competition between the S-cells and R-cells. The average number of S-cells in each R-cell neighborhood was higher in the random and uniform cases than in the clumped case ($N_{S\bar{R}}{\left(t\right)}^{c}<N_{S\bar{R}}^{r}\left(t\right)$,$N_{S\bar{R}}^{u}\left(t\right)$) (Fig 5D: yellow/orange boxplots vs. blue boxplots). The difference between the average number of S-cells in a neighborhood in the clumped case and those in the other two cases decreased from the 1st day to the 35th day (i,e., $N_{S\bar{R}}^{c}\left(1\right)∼N_{S\bar{R}}^{r}\left(1\right)>N_{S\bar{R}}^{c}\left(35\right)∼N_{S\bar{R}}^{r}\left(35\right)$,$N_{S\bar{R}}^{c}\left(1\right)∼N_{S\bar{R}}^{u}\left(1\right)>N_{S\bar{R}}^{c}\left(35\right)∼N_{S\bar{R}}^{u}\left(35\right)$). The higher number of S-cells in the VNHDs of the R-cells allowed the drug to free up sites more in the random and uniform cases than in the clumped case. In the first treatment break (from day 35 to day 95), the number of S-cells in the neighborhoods increased in all three cases due to the proliferation of S-cells during the “off” part of the treatment cycle (Fig 5D). Thus, the inhibition of growth of R-cells by S-cells was higher in the random and uniform cases than in the clumped case.

Finally, we quantified the strength of inter-species spatial competition (i.e., competition between R-cells). The average number of R-cells in a neighborhood in the clumped case was always higher than the numbers in the random and uniform cases ($N_{R\bar{R}}{\left(t\right)}^{c}>N_{R\bar{R}}^{r}\left(t\right)$,$N_{R\bar{R}}^{u}\left(t\right)$) (Fig 5E). Interestingly, the number of R-cells in each R-cell neighborhood in all three cases increased irrespective of drug administration because R-cells can proliferate regardless of drug administration. A greater number of R-cells in the neighborhood implies a stronger inhibition of R-cell growth by R-cells, leading to a slower rate of cell population growth (Fig 5B: slope of the total population growth in the clumped case < slope of the total population growth in the random and uniform cases).

In summary, in the random and uniform cases, the number of R-cells increased more quickly due to the space available in the neighborhoods, and it reached the level of 50% of the initial total cell population during the end of the second drug administration after the first “off” part of the treatment cycle (Fig 5B: dotted cyan line). Once the number of R-cells reached the level of 50% of the initial cell population, the ongoing (additional) cycle of treatment could not reduce the total population below the 50% level, leading to continuous treatment and a quicker progression (Fig 5B). Under CT-MTD, inter-species competition (R–R competition) was solely responsible for determining the TTP (higher competition leading to delayed TTP). Under AT, however, a combination of R–R competition and R–S competition seemed to determine the TTP. In other words, a more significant reduction in the growth inhibition of R-cells by S-cells combined with the increase in R–R competition drove a faster TTP in the random and uniform cases. In the clumped case, the R–R competition is the main determining factor of TTP.

### Clumped initial distribution results in higher clinical time gain (TG)

So far, we explored the impact of the initial R cell distribution on the AT and CT-MTD outcomes and investigated how the treatments modulate the inter- and intra-species competition (R–S and R–R, respectively), resulting in different treatment outcomes. During CT-MTD, the drug is supplied consistently without considering the response, which causes a prompt decline in the S-cell population and facilitates R-cell growth by lowering the local R–S spatial competition. On the other hand, during AT, the drug is supplied in short cycles to keep a tolerable number of S-cells, which are required in order to limit the R-cells’ growth by maintaining spatial competition. Therefore, AT is expected to maintain total cell growth for longer than CT-MTD. We quantified the benefits of AT over CT-MTD in terms of the TG (= Time to Tumor Progression(TTP) in AT—Time to Tumor Progression(TTP) in CT-MTD).

**Fig 6:**
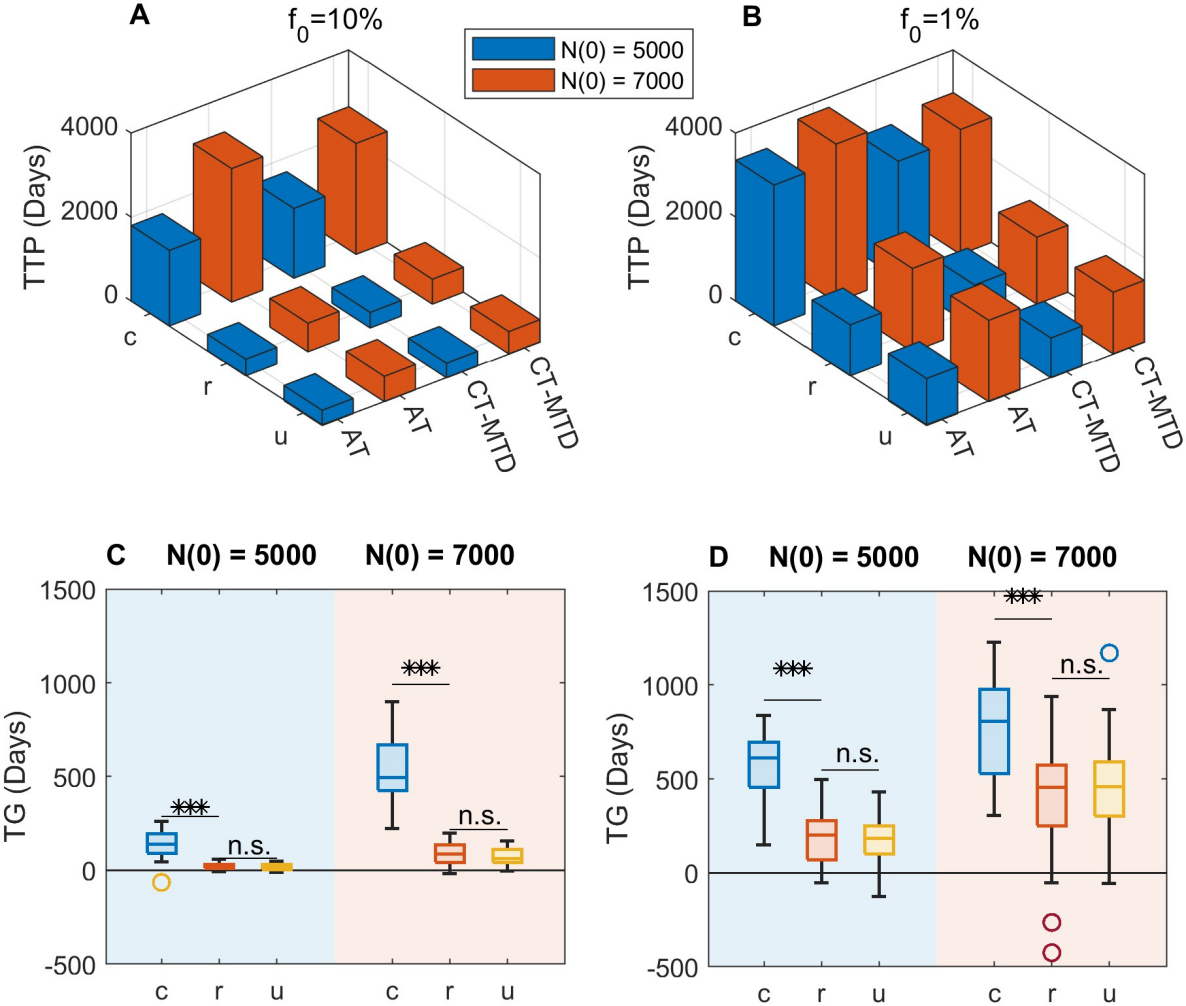
Role of the initial R-cell distribution on the benefit of AT over CT-MTD. (**A**) The blue and red bars show the TTP for *N*(0) = 5000 and 7000 respectively under both treatment strategies and different initial R-cell configurations of c (clumped), r(random) and u(uniform) for *f*_0_ = 10%. (**B**) Similar results to those in A are shown for *f*_0_ = 1%. (**C**) The boxplots show the TG in the 30 realizations for the clumped, random, and uniform initial cell configurations for *f*_0_ = 10% (***: *p* − *value* < 0.001; n.s.: not significant). The blue shaded and orange shaded regions show results for *N*(0) = 5000 and 7000 respectively. (**D**) Similar results to those in C are shown for *f*_0_ = 1%.

Fig 6 displays comparison of the two treatment outcomes of two different initial resistance (*f*_0_ = 10% and 1%) for two different initial number of cells (*N*(0) = 5000 and 7000) in three different initial R-cell configurations. Both for *f*_0_ = 10% (Fig 6A) and 1% (Fig 6B), we observe that TTP is longer for *N*(0) = 7000 than for *N*(0) = 5000, which is because the number of total cell population is higher for *N*(0) = 7000 that for *N*(0) = 5000. It is worth noting that in this study TTP is defined as the time when the total cell population reaches 120% of the initial cell population. Further, the average TTP is larger under AT than under CT-MTD for all initial settings, which shows that clumped initial cell configuration results in higher TG than the other types of cell configuration. However, AT extends the TTP 8.3, 5.4, and 5.1% for clumped, random, and uniform cases respectively concerned to the TTP under CT-MTD in the case of *N*(0) = 5000, *f*_0_ = 10%. A similar percentage-wise increase is observed for other initial conditions as well.

On the other hand, in terms of absolute change, the benefit of AT is greater if a smaller fraction of resistance was present initially (Fig 6C vs. Fig 6D). Comparing the effect of initial R-cell configuration, a clump (*c*) initial distribution results in significantly higher TG in all the cases (Fig 6C and 6D, *p* – value < 0.001). Interestingly, its expected time gain relative to the random configuration is longer for higher initial resistance. For *f*_0_ = 10%, clumped initial R-cell distributions result in more than 6 times higher TG compared to random R-cell distributions. For *f*_0_ = 1%, clump distributions lead to about 2 − 3 times higher TG.

Tumor carrying capacity [42, 43] and migration rates [44] may relax the competition and promote the tumor growth. An increasing carrying capacity leads to reduced local competition between cancer cells. Thus, the benefit of adaptive therapy decrease (S2 Fig). In addition, CAFs provide growth factors for tumor cells, leading to local competition alteration [30]. In the next two subsections, we discuss the consequence of cancer cell migration and fibroblast-mediated growth on the treatment outcome. The uniform and random cases did not show significant differences in terms of TG. Furthermore, the clumped case and random case were two extreme versions of similar types of distributions. Thus, we focused on the case of clumped R-cell distribution in the next two subsections.

### Increased cell migration lead to less benefit of AT over CT-MTD

**Fig 7:**
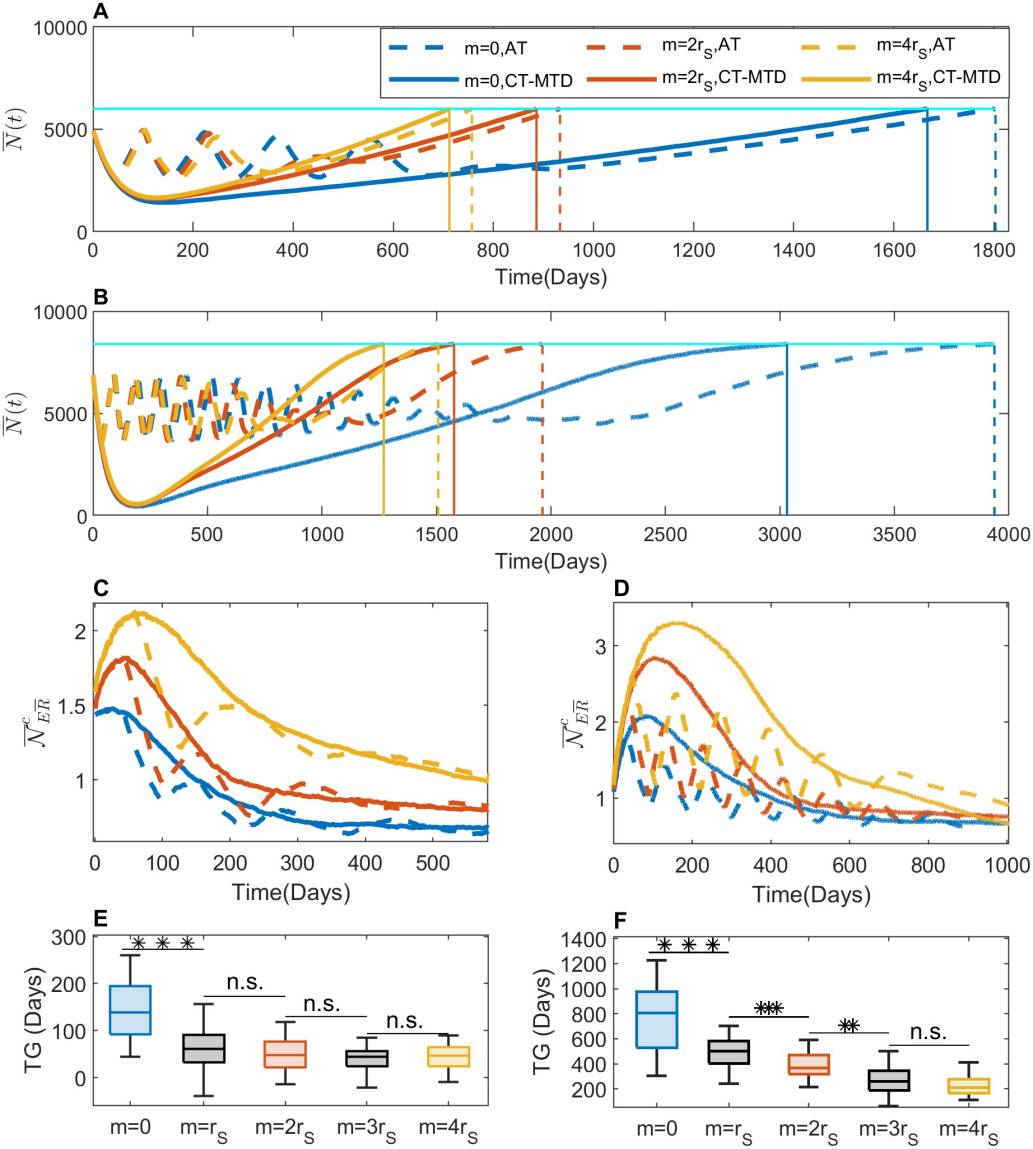
Effects of cell migration on treatment responses. (**A**) The time evolution of the mean of the total cell population ($\bar{N}\left(t\right)$) in the 30 simulations is shown for *m* = 0 (blue), 2*r*_*S*_ (red), and 4*r*_*S*_ (yellow) under AT (dashed line) and CT-MTD (solid line) with *N*(0) = 5000, *f*_0_ = 10%. The vertical solid lines show the time to tumor progression (TTP) under CT-MTD. The vertical dashed line shows the TTP under AT. The horizontal cyan line shows the 120% level of the initial cell population (progression threshold). (**B**) The same result as in A is shown for *N*(0) = 7000, *f*_0_ = 1%. (**C**) The time evolution of the mean of the average number of empty sites in the VNHD of each R-cell in the 30 realizations (${\bar{N}}_{E\bar{R}}^{i}\left(t\right)$) is shown with the same line styles as in A. (**D**) The same result as in C is shown for *N*(0) = 7000, *f*_0_ = 1%. (**E**) The boxplots of the TG for *m* = 0, *r*_*S*_, 2*r*_*S*_, 3*r*_*S*_, 4*r*_*S*_ are shown. (**F**) The same result as in D is shown for *N*(0) = 7000, *f*_0_ = 1%.

To investigate the impact of cell migration on therapeutic responses, we simulated our model for different values of migration rates, *m*. In this study, the simulation time unit is cell doubling time (1 day). For simplicity, cell growth rates and death rates were assumed to be multiple of growth rate of the drug-sensitive cell population (*r*_*S*_) [16, 27]. To be consistent, we assume cancer cell migration rates as multiple of cell growth rate (*m* = 0, *r*_*S*_, 2*r*_*S*_, 3*r*_*S*_ or 4*r*_*S*_) as well [45, 46]. Under both AT and CT-MTD, temporal changes of tumor volume are shown in Fig 7A and 7B for *N*(0) = 5000, *f*_0_ = 10% and *N*(0) = 7000, *f*_0_ = 1% respectively. Increased cell migration promoted faster cell population growth, leading to a shorter TTP. Fig 7A shows that for *N*(0) = 5000, *f*_0_ = 10% the time to progression without cell migration was 1667 days under CT-MTD. The TTP decreased to 884 days when the cell migration increased to 2 times, and further decreased to 712 days when the rate was 4 times. On the other hand, the time to progression without cell migration was 1803 days under AT. The TTP decreased to 933 days when the cell migration increased to 2 times, and further decreased to 757 days when the rate was 4 times. Similar scenario is observed for *N*(0) = 7000, *f*_0_ = 1% (Fig 7B).

To understand the mechanism by which migration causes a faster relapse, we investigated the temporal evolution of the local growth capacity (number of empty sites in the VNHD of each R-cell; ${\bar{N}}_{E\bar{R}}^{c}\left(t\right)$) (Fig 7C and 7D). The figure shows that ${\bar{N}}_{E\bar{R}}^{c}\left(t\right)$ was smaller for *m* = 0 than for *m* = 2*r*_*S*_ and 4*r*_*s*_ under CT-MTD. We observed a similar impact of cell migration on the AT response. During the “on” period of the treatment, the S-cells died, resulting in an increase in the number of empty sites in the VNHD of each R-cell in all of the migration rate cases. The lowest increase, however, was observed in the absence of migration. This increase in empty sites in each R-cell’s neighborhood as a result of cell migration implies a higher growth potential for each R-cell, leading to a faster treatment failure. Therefore, cell migration reduces the local spatial competition, leading to a rapid increase in the total cell population.

During the first treatment cycle on AT (Fig 7A and 7B), a higher cell migration delays the treatment vacation, which allows the R-cell population to grow more than it grows in the absence of migration. Similar delays in subsequent treatment vacations due to a higher migration rate allows R-cells to grow further due to relaxed cell competition and results in a fewer number of treatment cycles (e.g., 4 cycles of blue dashed line vs. 2 cycles of yellow dashed line in Fig 7A). Therefore, a higher cell migration relaxes competition and shortens the TTP under AT more than that under CT-MTD, which results in a reduction in the time gain due to migration. Fig 7E and 7F show the TG for *N*(0) = 5000, *f*_0_ = 10% and *N*(0) = 7000, *f*_0_ = 1% respectively. The result shows that the effect of a higher cell migration is more significant when initial resistance is low (statistically significant between *m* = 2*r*_*S*_ and *m* = *r*_*s*_, between *m* = 3*r*_*S*_ and *m* = 2*r*_*s*_ in Fig 7F).

### Cancer associated fibroblast-mediated drug resistance

So far, we investigated the role of initial R-cell distribution, carrying capacity, and cell migration on the therapeutic responses. Cancer-associated fibroblasts (CAF) are known to promote cancer cell growth and drug resistance [29–33]. In particular, recent studies revealed the impact of fibroblast location on the outcomes of continuous therapy [30, 34, 47]. The distribution of CAFs in real tumor tissues is diverse, which is difficult to categorize [32, 35–37]. As a proof of principle, we simply considered five representative distributions of fibroblast (defined in the section Initial and boundary conditions), which though cannot be exactly mapped to a real tumor micro-environment, could help gain a theoretical understanding and infer plausible outcomes in real-life scenario. To be more specific, we compared the cases of FC (CAFs overlapping with the R-cell clump in the center) and FCp (CAFs partially overlapping with the R-cell clump) with NoF (no CAFs) to understand the impact of the *relative positioning* of the CAFs with the R-cell clump. Next, we compared FSq (CAF surrounding the R-cell clump) and FSp with FCp (CAFs over a “L” shaped region, partially surrounding the R-cell clump) to explore the impact of *shape and orientation* of CAFs with respect to the R-cell clump. Finally, we compared FR (randomly distributed CAFs) with FCp to understand the impact of segregated CAF region.

**Fig 8:**
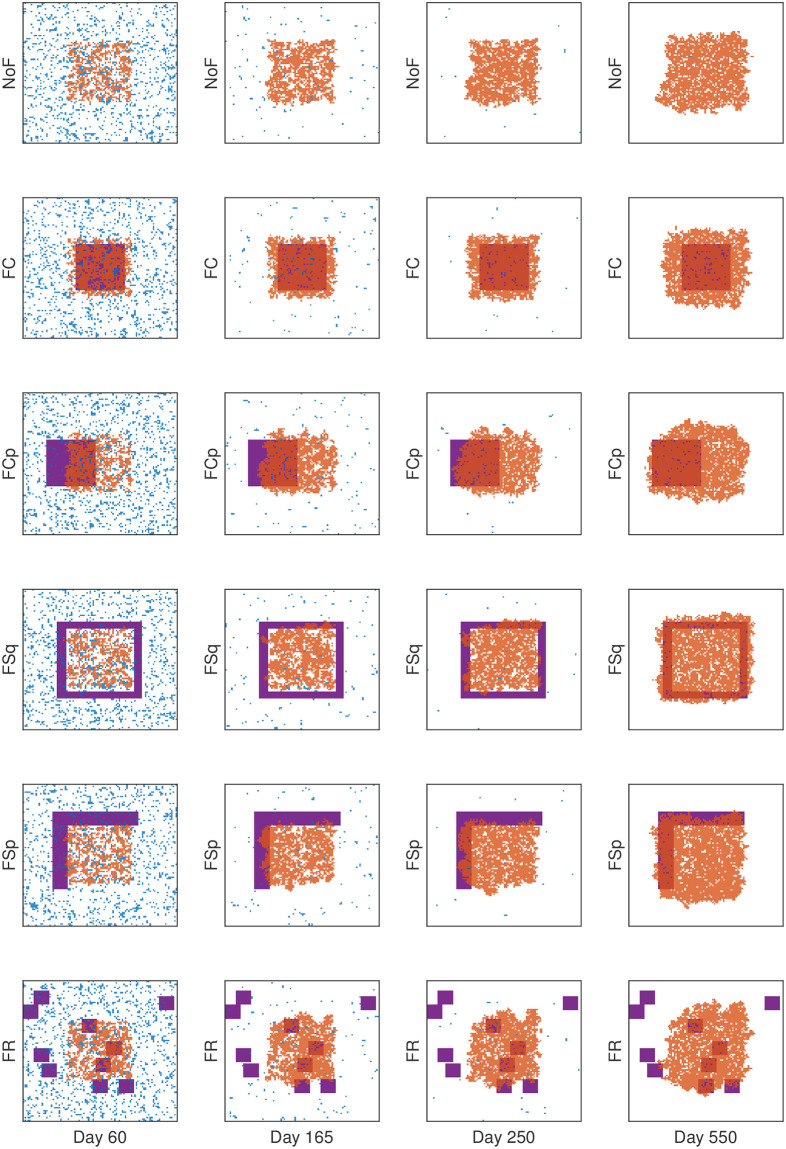
Cell configurations for CAF-mediated tumor growth. Cell configurations at different times under CT-MTD (continuous MTD therapy) for all type of fibroblast configurations. Each row shows results for one type of fibroblast configuration. The blue and orange dots show the S-cells and R-cells respectively and the purple shaded areas show the fibroblast regions. Specification of the fibroblast region is shown in the section Initial and boundary conditions.

Fig 8 shows the temporal change of cell configuration with different type of CAF distributions under CT-MTD, assuming the growth promotion rate by CAF to be two-fold (*r*_*iF*_ = *αr*_*i*_, where *i* ∈ {*S*, *R*}, where *α* = 2). Here, we explain the impact of each CAF configuration compared with the no fibroblast case (row 1). In row 1 (NoF), temporal change in cell configuration is shown in absence of fibroblast. In row 2 (FC), most of the R-cells reside in the CAF region. On days 60 and 165, we observe cell density is slightly higher in the case of FC than in the case of NoF. In both cases, the growth of the R-cell clump is almost radially symmetric (Please refer to column 1 and 2 of S2 Movie). Comparing row 3 (FCp) with row 1 (NoF), we observe that the cell growth is skewed towards the left due to the CAF location(Please refer to column 3 of S2 Movie). In row 4 (FSq), the outer cells in all directions grow at a faster rate due to the CAF-mediated advantages, which are almost radially symmetric as the CAF fuel the growth from all directions (Please refer to column 4 of S2 Movie). However, in the case of FSp (row 5), cells in the left and upper directions get the benefit of CAF-mediated growth. We observe that on days 165 and 250 cells are growing more in the direction of CAF than in the other two directions (Please refer to column 5 of S2 Movie). In the case of FR (row 6), the three clumps of fibroblast near the center accelerated the tumor growth initially. As the R-cell clump grows, it gets access to other clumps momentarily (Please refer to column 6 of S2 Movie).

**Fig 9:**
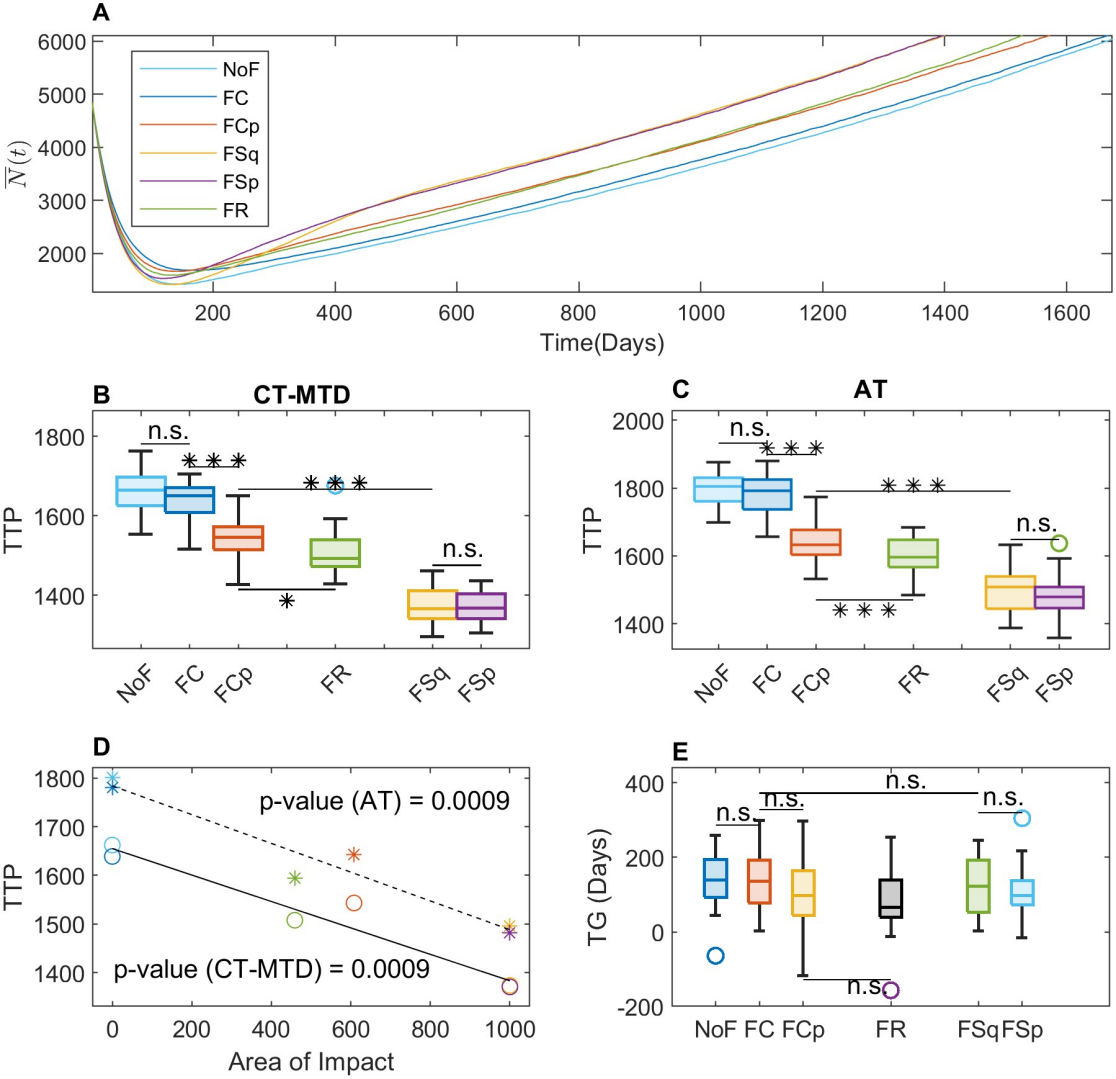
Consequences of CAF-mediated resistance with clumped initial R-cell distribution. (**A**) The time evolution of the average of the total cell population ($\bar{N}\left(t\right)$) in the 30 simulations is shown for CT-MTD for all types of fibroblast configurations. NoF: no fibroblast, FC: fibroblast overlapping with the R-cell clump at the center of domain, FCp: fibroblast partially overlapping with the R-cell clump at the center, FSq: fibroblast completely surrounding the R-cell clump. FSp: fibroblast over a “L” shaped region, partially surrounding the R-cell clump, FR: fibroblast clumps randomly placed in the domain. (**B**) Boxplot of the time to progression (TTP) in the 30 realizations under CT-MTD. (**C**) Boxplot of the time to progression (TTP) in the 30 realizations under AT is shown. (**D**) Correlation of TTP with the area of impact is shown. The circles and the asterisks (the colors are similar to the legend in A) show the TTP under CT-MTD and AT respectively. The solid and dashed lines are the respective regression lines (sold line: CT-MTD, dashed line: AT). The *p* − *values* for AT and CT-MTD indicate significance of the correlation. (**E**) Time gain (TG) is shown as boxplots for all six types of fibroblast distributions.

To get a comprehensive understanding of tumor cell growth dynamics, we consider temporal evolution of total tumor cell populations for all CAF-distributions (Fig 9A) under CT-MTD. For all the distributions, the total cell population decrease to a minimum and then grows back. To explore the impact of the relative positioning of the CAFs, first, we compare the cases FC and FCp with NoF. The total cell population decreases to the minimum (137, 154, and 177 cells in the cases of FCp, NoF, and FC, respectively (Fig 9A and S2 Movie)). In the first 100 days the average decreasing rates of cell population are about 31.5, 32.5, and 34.7 *cells*/*day* for FC, FCp and NoF respectively, which follow the order of the initial number of R-cells in CAF region. Higher the number of R-cells in the CAF region initially, lower the decreasing rate. For FC, almost all of the R-cells initially reside in the CAF region (Fig 8: row 2), while in the case of FCp, it is a fraction (less than 50%) of the R-cells. Thus, the growth of more R-cells is promoted by the CAFs, and the cell population decreases less rapidly with FC than in the other two cases.

Once all of the sensitive cells are eradicated by CT-MTD and more resistant cells grow and saturated the CAF-region, the growth dynamics of the total cell population in NoF and FC become similar because of the elevated growth rate due to the presence of the CAF at the center had no significant impact due to higher R-R spatial competition (Fig 8: column 3,4), which results in no significant change in TTP (TTP in NoF vs. TTP in FC in Fig 9B). It is worth noting that when the effect of CAF was increased (*α* = 4), TTP with FC significantly decreased from TTP in NoF case (*p* − value < 0.01, S4B Fig). In the case of FCp, the R-cells on the leading edge of the clump keeps getting benefited until the population pass over the CAF region (Fig 8: row 3). The average growth rates of the tumor between the day 200 to 550 are 2.9 and 2.2 *cells*/*day* for FCp and FC respectively, i.e., the partially overlapping position of the CAF in the case of FCp promoted the tumor growth at a slightly higher rate. This is also visible in the third row of Fig 8, that on the day 550, the R-cell clump is larger than that in the case of FC and skewed towards the location of the CAFs. Tumor cells with FCp distribution results in a quicker progression than the other two cases (*p* − value < 0.001, Fig 9B and S4B Fig).

Second, we compare the cases FSp and FSq with FCp, to explore the scenario when the CAF region is stretched out and aligned with the boundary of the R-cell clump. Initially, none of the R-cells is in the CAF region in the cases of FSp and FSq (unlike FCp). The total cell population decreases more rapidly in these cases than in the case FCp (Fig 9A). The average decreasing rates until day 100 are 35.2, 34.5 and 32.5 *cells*/*day* respectively for FSq, FSp and FCp respectively. Once the growing R-cell clump reaches the CAF region, the leading R-cells start growing at a higher rate due to the CAF-mediated growth advantage and scarce local competition. The average growth rates of the tumor between the day 200 to 550 are 4.6, 4 and 2.9 *cells*/*day* respectively for FSq, FSp and FCp respectively, resulting in significant decrease in TTP in both the cases(*p* − value < 0.001, Fig 9B and S4B Fig). Since leading cells from two directions gain CAF mediated benefit in FSp, the cell population grows faster (Fig 8: row 5) than in the case FCp. For FSq, the leading R-cells of four sides start receiving the benefit of the CAF. When the CAF-mediated effect was increased, we observed similar growth dynamics in the S4B Fig.

Finally, we compare the cell population growth in the case of FR with FCp. Our simulation shows that the cell population grows steadily at a slightly higher rate than in the case of FCp (Fig 9A). The average growth rate from day 200 to day 1400 is 3.1 and 3.2 for FCp and FR respectively. For FR, a fraction of the leading cells get access to the randomly scattered CAF regions momentarily and gain the CAF-mediated growth advantages in absence of R-R or R-S competition (Fig 8: row 6). Consequently, FR results in lower TTP than FCp (Fig 9B and S4B Fig).

A similar type of comparative scenario is observed for tumor growth under AT for *α* = 2 and 4 (Fig 9C and S4C Fig respectively). From the above results, we observe that CAFs outside the central R-cell clump could accelerate tumor progression. However, if the CAFs are located so far from the central clump that the cells reach the CAF region after progression, the CAF will not have any impact on TTP. In this study, we assume that tumor progression occurred when the total cell population reaches 120% of the initial number of cells. The progression occurred when tumor cells reaches 10 lattice points inside from the boundary in our computational domain (solving for P in (*L* − 2 × *P*)/*N*(0) ≈ 1.2, where *L* = 100 and *N*(0) = 5000, then *P* = 10). We name the area of the CAFs in this region as the *Area of Impact*. The area of impact for each type CAF distribution is shown in S3 Fig. Fig 9D and S4D Fig shows that the TTP is highly correlated with the area of impact under both type of treatment strategy for *α* = 2 and 4 respectively. However, in terms of TG, no significant impact was observed. Heterogeneity of CAF distributions does not significantly enhance the performance of AT over CT-MTD.

**Fig 10:**
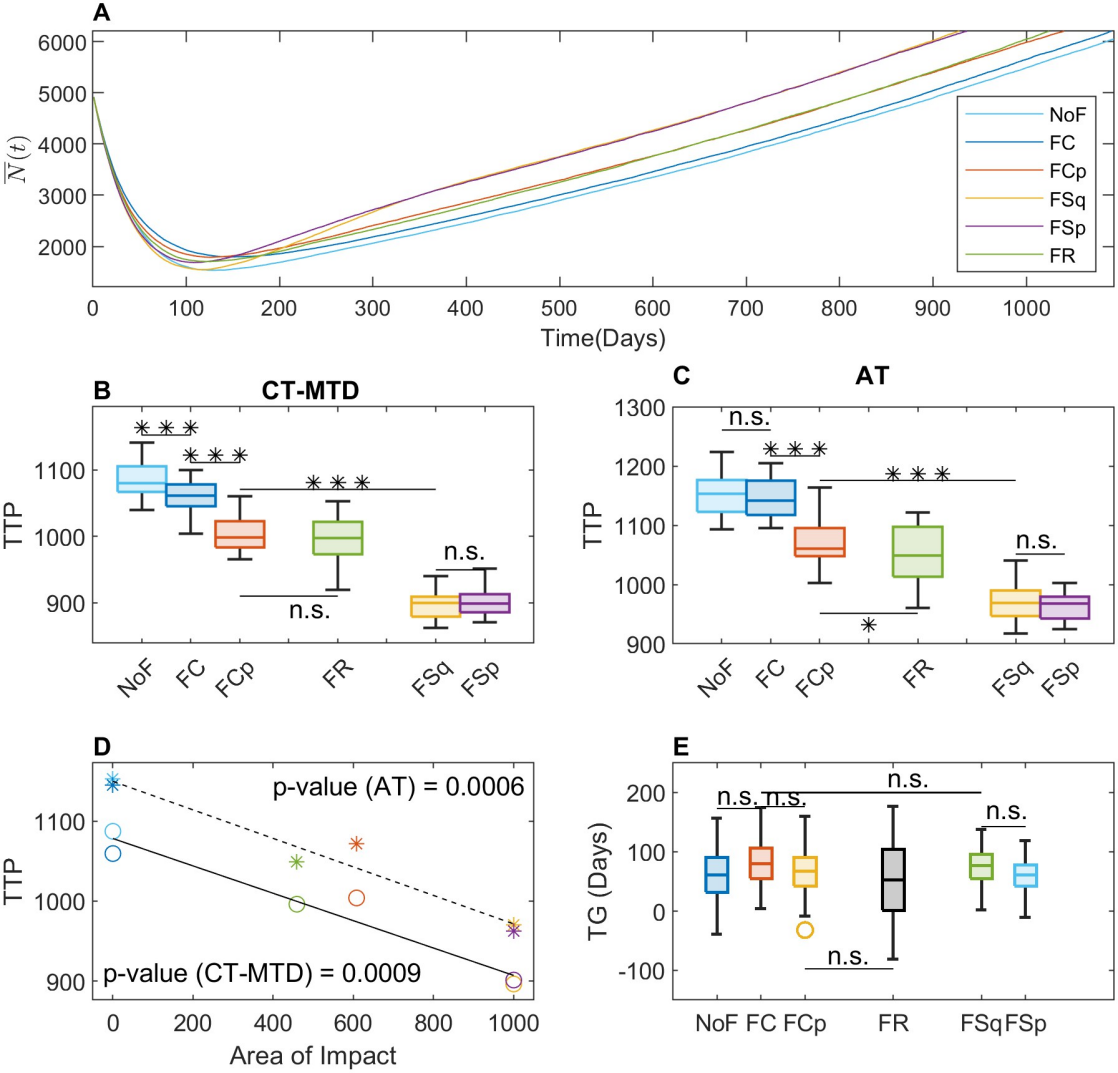
CAF-mediated growth with clumped initial R-cell distribution and cell migration (*α* = 2, *m* = 1). (**A**) The time evolution of the average of the total cell population ($\bar{N}\left(t\right)$) under CT-MTD in the 30 simulations is shown for all types of CAF configurations. (**B**) Boxplot of the time to progression (TTP) under CT-MTD in the 30 realizations. (**C**) Boxplot of the time to progression under AT in the 30 realizations. (**D**) Correlation of TTP with the perimeter of impact. The circles and the asterisks (the colors are similar to the legend in A) show the TTP under CT-MTD and AT respectively. solid line: CT-MTD and dashed line: AT regression line. The *p* − *values* for AT and CT-MTD indicate significance of the correlation. (**E**) Time gain (TG) is shown as boxplots for all type of CAF distributions.

To explore the impacts of both cell migration and fibroblast location on therapeutic outcomes, we additionally simulated the model with the clumped R-cell distribution and a cell migration rate of *m* = 1 (Fig 10). The result was similar to that of the case with the clumped initial distribution and *m* = 0 (Fig 9). However, as we observed before (Fig 7), due to the reduction in R–R competition as a result of cell migration, tumor progression occurred earlier for all types of CAF-configurations and treatment strategies of adaptive therapy (AT) and continuous MTD therapy (CT-MTD).

### Adaptive therapy on a virtual patient with multiple metastatic lesions: Three detected lesions and one undetected lesion at the beginning of therapy

**Fig 11:**
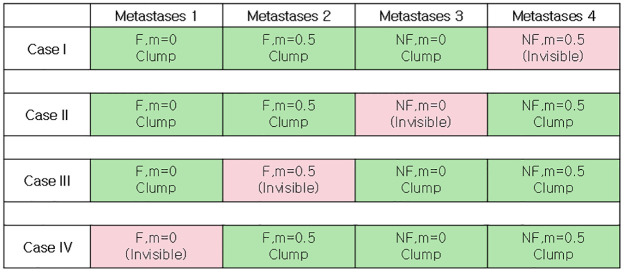
Combinations of the four metastasis scenarios. The green and red colors correspond to detected and undetected metastases, respectively. F and NF correspond to the existence and absence of fibroblasts, respectively.

**Fig 12:**
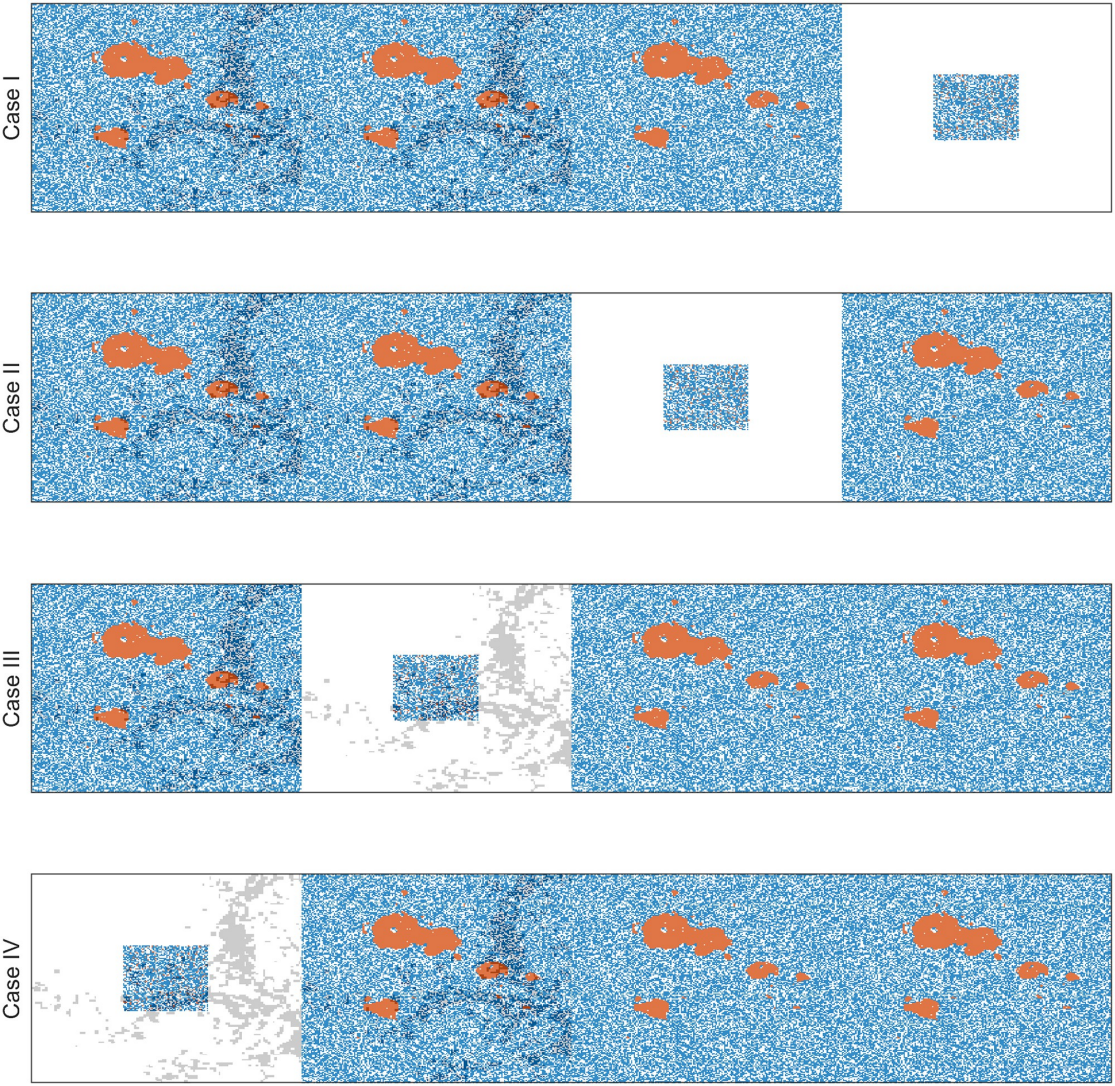
Initial cell configurations for the four cases with an invisible clumped metastasis. The red, blue, and white dots correspond to R-cells, S-cells, and empty sites. The gray dots shows the sites accompanied by CAFs.

**Fig 13:**
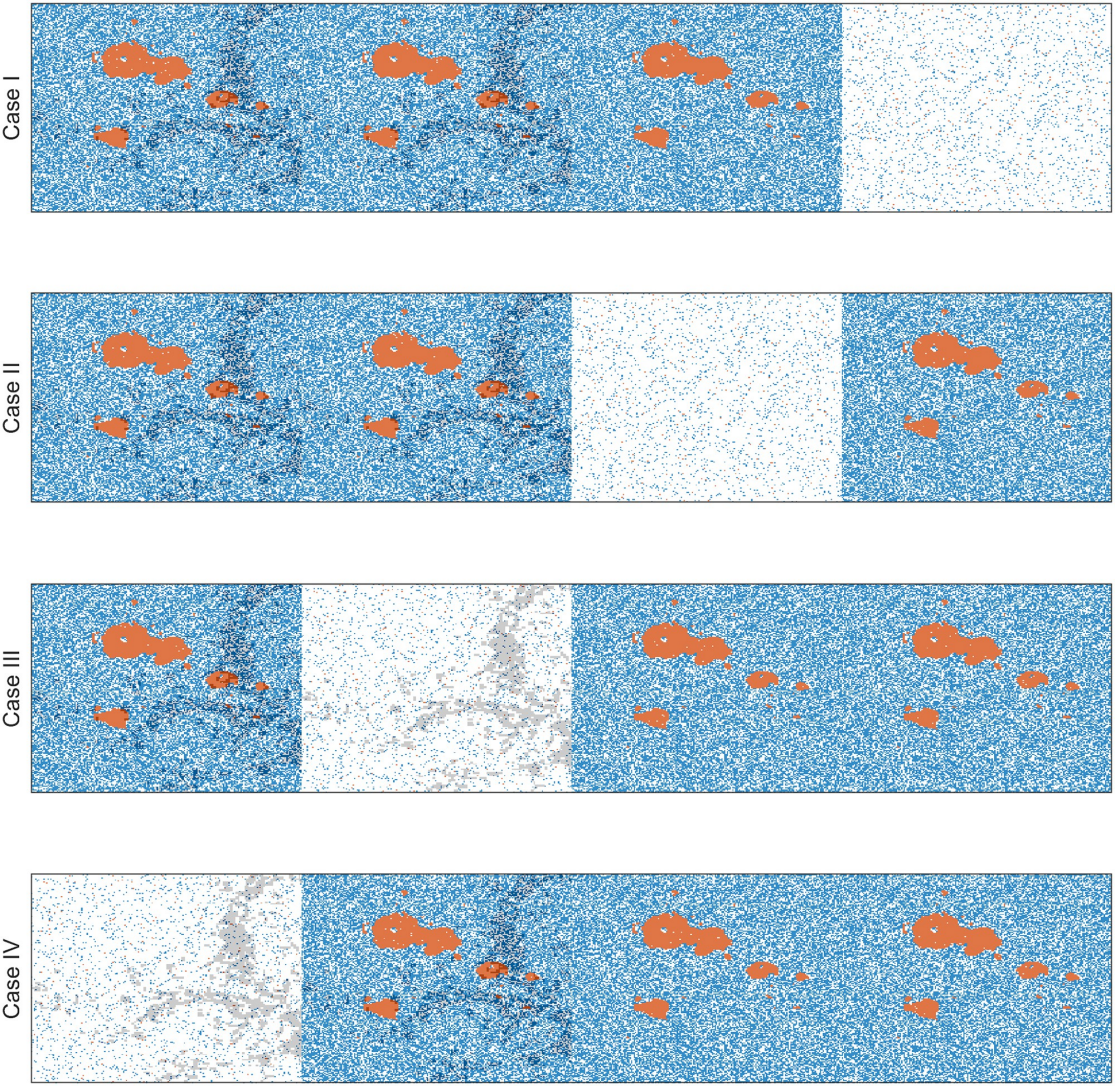
Initial cell configurations for the four cases with a random invisible metastasis. The red, blue, and white dots correspond to R-cells, S-cells, and empty sites. The gray dots show the sites that are accompanied by CAFs.

In the sections above, we investigated the treatment response with a single tumor lesion (either a primary or metastatic site). Patients with advanced cancers who undergo the systematic therapy that we consider in this study typically present with multiple metastases. To understand the impact of the spatial heterogeneity of R-cells and CAF on treatment outcomes, we simulated AT and CT-MTD in a virtual patient with four metastatic lesions, each of size 200 × 200. Each metastatic lesion had its own independent domain, in which the cells were subject to space constraints. However, all metastatic lesions were subjected to the same systemic treatment, which was guided by a systematic biomarker that was represented by the total number of cells in all metastatic lesions. The characteristics of the local microenvironments were significantly different. For instance, the numbers of CAFs were different among the metastatic sites. Due to the different compositions and densities of extracellular matrix, tumor cell migration can be different. As a proof of concept, we considered four combinations, which are shown in Fig 11, and considered a tumor consisting of four metastases that held the four different biological combinations. In addition, we assumed that one of the metastases was invisible (contained too few cells to be detected initially). We assumed that the number of tumor cells in the invisible metastatic site was 10% of number of cells in the other metastases. Therefore, we modeled four different cases of tumors with four metastases, of which one metastatic lesion was invisible (presented by the red color in Fig 11). For the visible metastases, we assumed a clumped initial R-cell distribution. We also assumed that the total number of cells was 10 times the number of R-cells (*N*(0) = 10*R*(0)) in each of the metastases. The S-cells were randomly distributed over each metastatic lesion’s domain. The locations of the CAFs were assumed to be scattered. We simulated these four metastatic tumor models for two types of invisible metastases: (i) All the tumor cells belonged to a 60 × 60 grid centered with the metastases (clumped), or (ii) all of the tumor cells were sparse over the all of the metastases (random). In both cases, the R-cells made up 10% of the total cell population and were dispersed randomly over the respective areas. Figs 12 and 13 show the initial cell configurations of the four metastatic lesions in the four cases mentioned (in Fig 11) for the clumped and random invisible metastases.

In these simulations, we use two different criteria for tumor progression: emergence time (ET) and TTP. ET was defined as the time for a new metastatic site to be detected, which was assumed to be the time when the total cell population was 50% of the overall domain’s carrying capacity in the respective metastasis. The TTP was defined as the time when the total cell population of the four metastases reached 120% of the total initial cell population.

**Fig 14:**
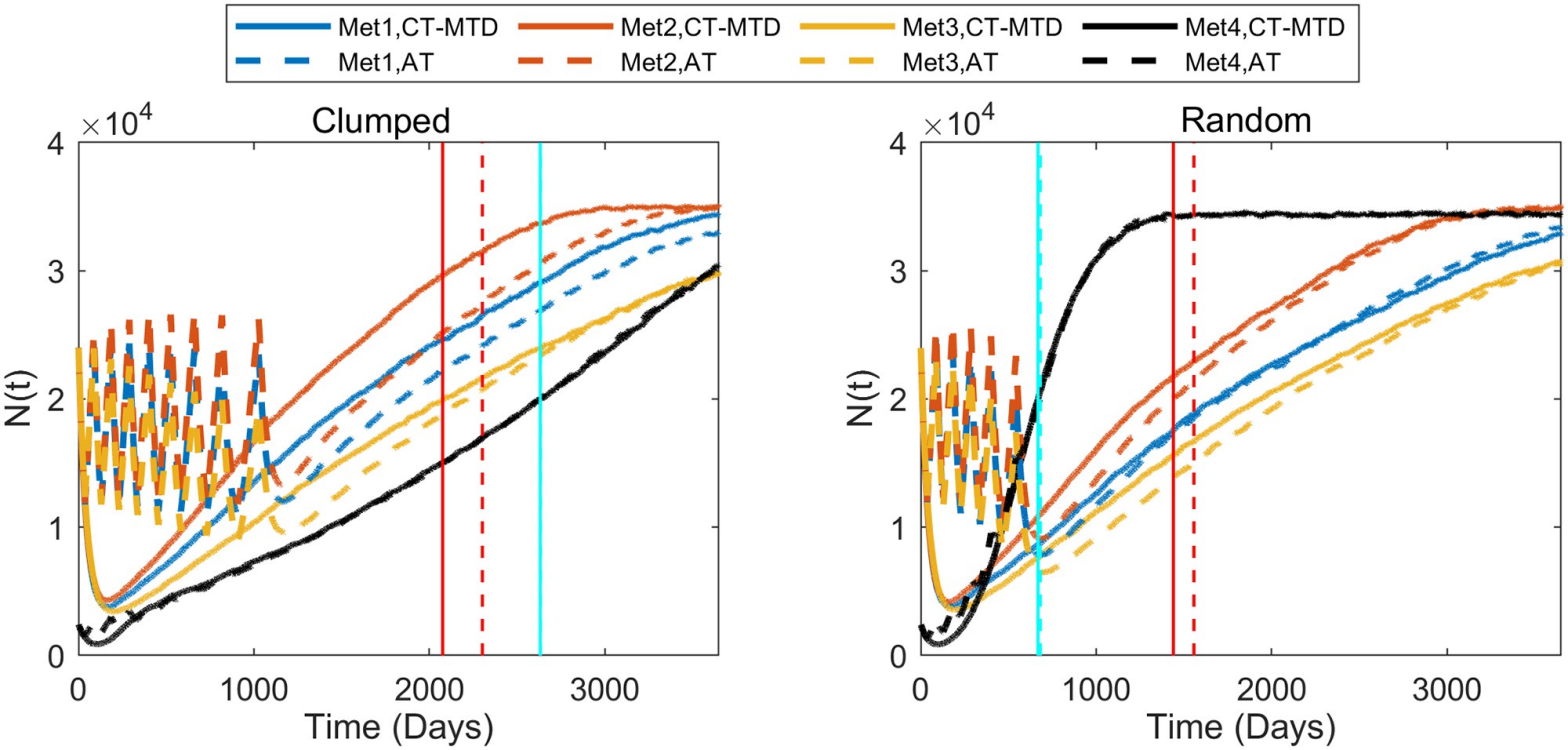
Complex dynamics of multiple metastases under AT and CT-MTD. The time evolution of the total cell population in the four metastases is shown in the sub-figures for Case I. The first and second columns show the results for the initial clumped and random cell configurations in the invisible metastasis, respectively. In each sub-figure, the blue, red, yellow, and black colors show the total cell populations in metastasis 1, metastasis 2, metastasis 3, and metastasis 4, respectively; the vertical cyan lines show the emergence time (ET) of the invisible metastasis, and the red line shows the TTP. The solid and dashed lines show results under CT-MTD and AT, respectively.

**Fig 15:**
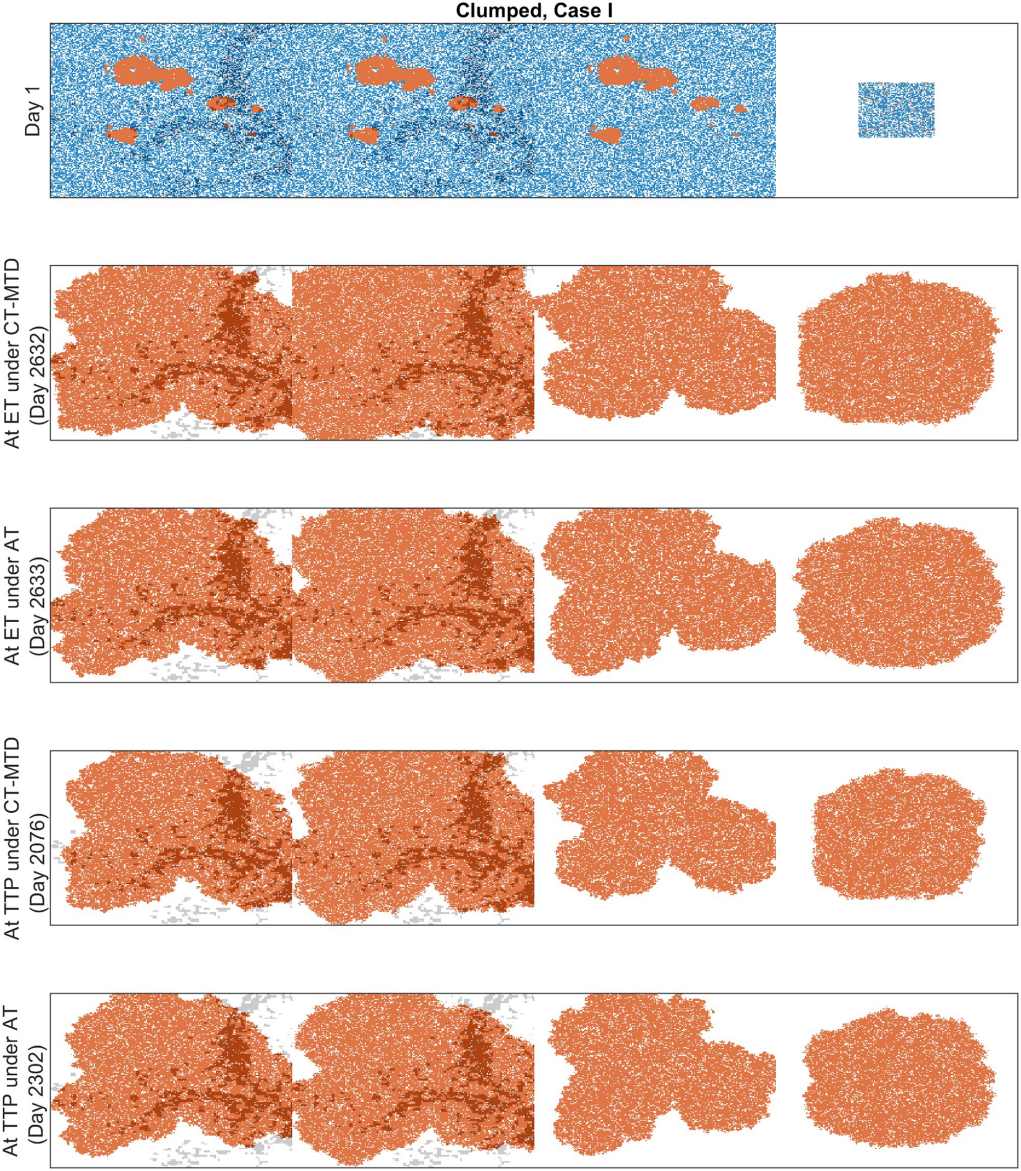
Cell configurations at the ET and TTP under CT-MTD and AT for Case I with the clumped invisible metastasis. The red, blue, and white dots correspond to R-cells, S-cells, and empty sites. The gray dots show the sites that are accompanied by fibroblasts.

**Fig 16:**
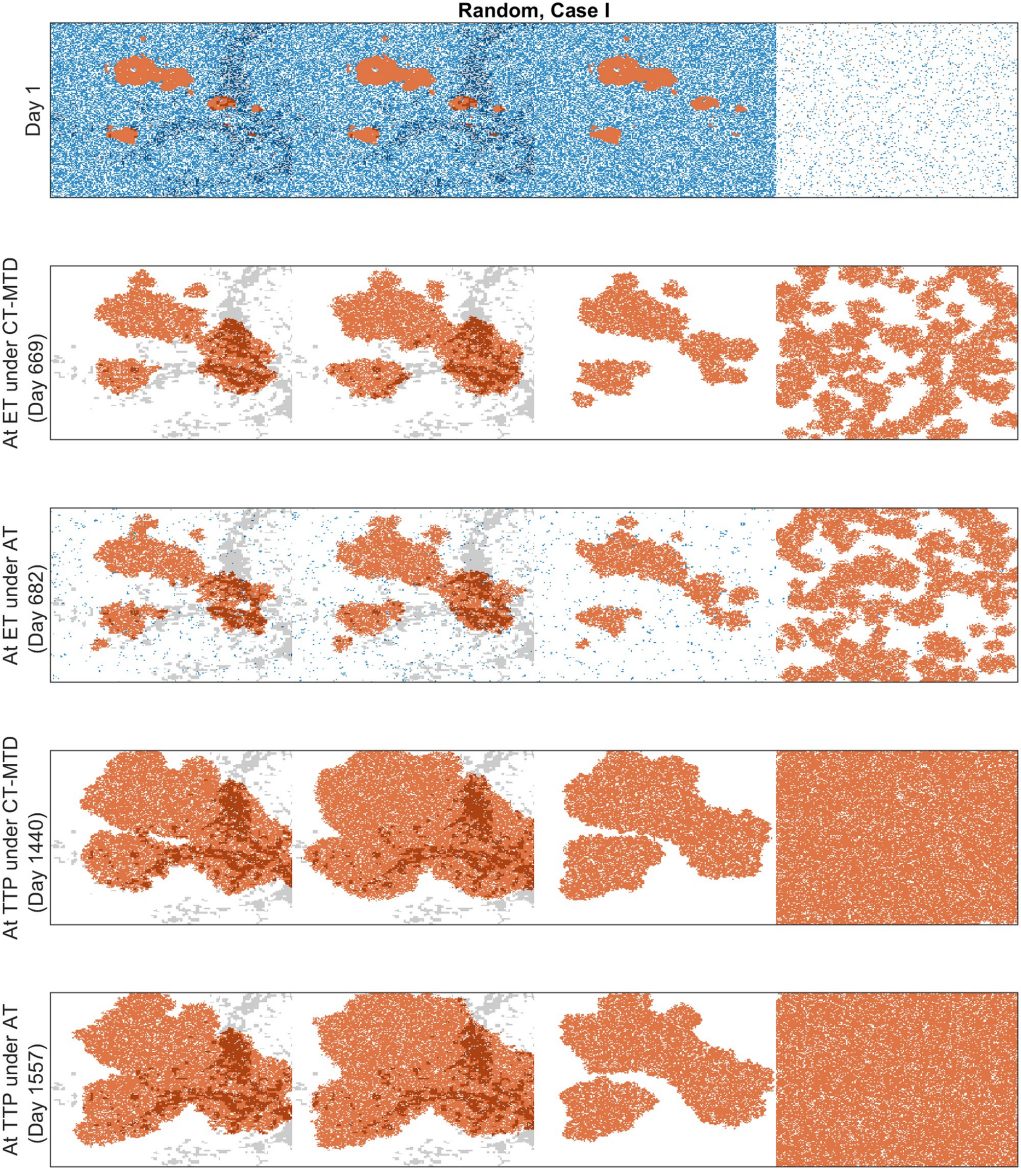
Cell configurations at the ET and TTP under CT-MTD and AT for Case I with the random invisible metastasis. The red, blue, and white dots correspond to R-cells, S-cells, and empty sites. The gray dots show the sites that are accompanied by fibroblasts.

We observed that both cell migration and CAFs can promote faster relapses (shorter TTP) in Sections “Increased cell migration lead to less benefit of AT over CT-MTD” and “Cancer associated fibroblast-mediated drug resistance”. Similar consequences were observed here. For instance, in case I with the clumped invisible metastatic lesion (Fig 14, first graphs in the left column), the total cell population grew faster in metastatic lesion 2 than in metastatic lesion 1 (Fig 15) due to the higher cell migration probability in metastasis 2. The total cell population grew faster in metastasis 1 than in metastasis 3 (Fig 15) due to the fibroblasts in metastasis 1. The invisible metastasis (metastasis 4) become noticeable on day 2632 under CT-MTD and on day 2633 under AT (Fig 15, rows 2 and 3, respectively) when the total number of cells in the fourth lesion reached 50% of the domain carrying capacities of those specific metastases. The ET was almost the same for CT-MTD and AT (vertical solid cyan line vs. vertical dashed cyan line), but the TTP in CT-MTD was shorter than that in AT (solid (CT-MTD, 2076 days) and dashed (AT, 2302 days) red lines). The cell configurations are shown in the fourth and fifth rows of Fig 15. Most importantly, when tumor progression had already occurred, the invisible metastasis had not yet reached a detectable tumor size. Please refer to S3 Movie for a continuous illustration of the phenomena discussed above. We observed a similar order in the growth of the tumor cell population in metastases 1 to 3 in Case I, as well with the random invisible metastasis (Fig 14, left vs. right figures). The cell configurations at crucial times are shown in Fig 16 and a continuous illustration is provided in S4 Movie. The cell growth in the random invisible metastasis was much faster than in all other metastases, in agreement with the results in sections “Impact of the initial R-cell configuration on the TTP under CT-MTD” and “Impact of the initial R-cell configuration on the TTP under AT”. Importantly, the resistant cell populations in this metastatic lesion experienced less competition with the sensitive cell population because the duration of the systematic therapy determined by the sum of all metastatic lesions was so long that most sensitive cells in the lesion were killed off by the first cycles. The random distribution imposed less competition between the resistant cell populations, resulting in the rapid growth of resistant cells. The fourth invisible metastasis became the largest on day 399 under CT-MTD and on day 574 under AT.

**Fig 17:**
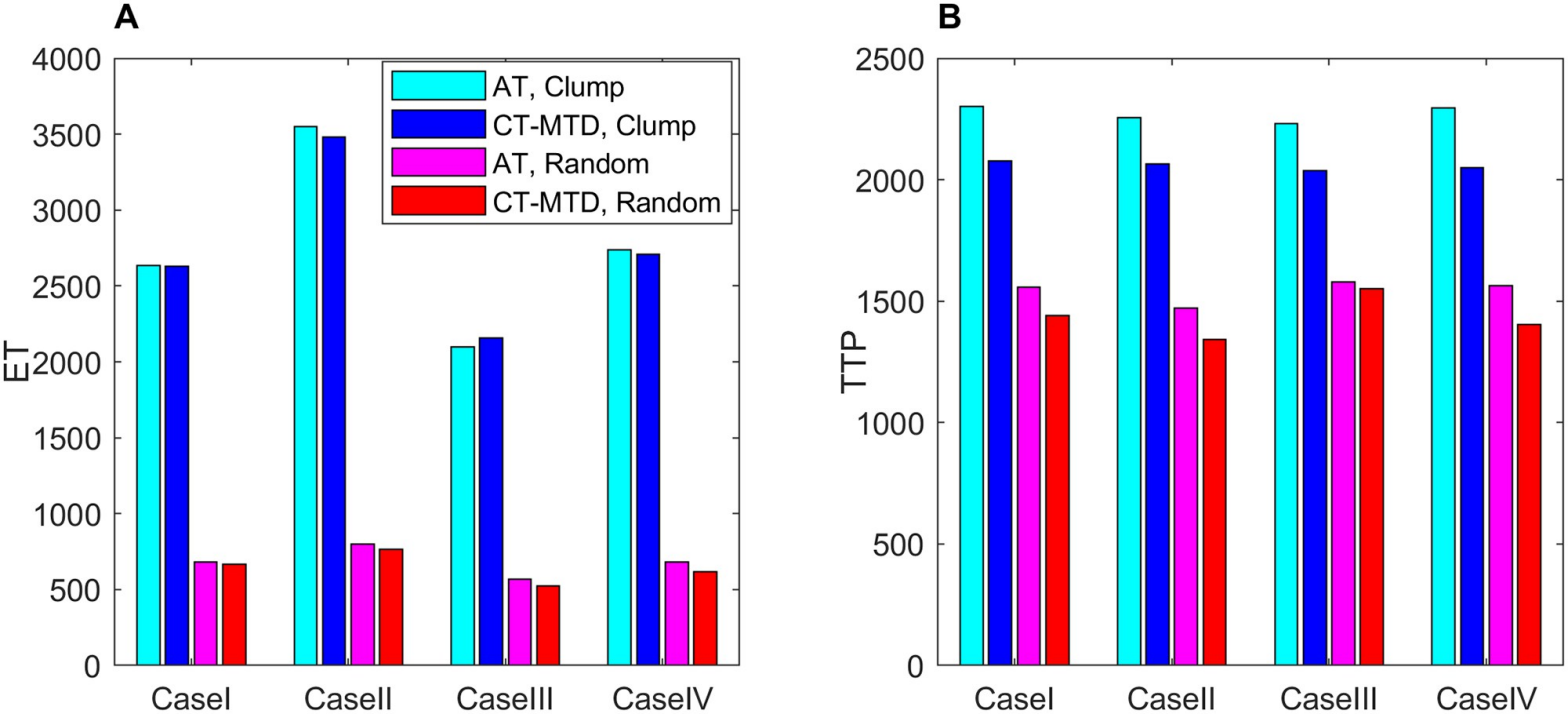
Bar chart of the ET and TTP under CT-MTD and AT for Cases I to IV. (**A**) clumped invisible metastases, (**B**) random invisible metastases.

For Cases II, III, and IV, similar results were obtained (S5 Fig). A comparison of the ET and TTP in the four cases is shown in Fig 17. The ET was more delayed in Case II than in Case I, as the higher cell migration in Case I led to a faster expansion of the tumor. The growth of the invisible metastasis was the fastest in Case III due to presence of CAFs and the higher cell migration rate. The growth of the invisible metastasis in Case IV was slower than in Case III due to the lack of migration. However, the TTP did not follow this ordering, as the TTP depends on the total number of cells in all of the metastatic sites.

## Discussion

Adaptive therapy has been shown to offer delayed progression with a lower cumulative dose rate by exploiting competition between tumor cells [16]. Within tumor tissues and throughout normal tissues, cells compete for space and survival with their neighbors. As recent studies have demonstrated, the spatial structure can shape a tumor’s evolution [19, 26, 27, 48]. This spatial competitive aspect has been further experimentally investigated [26, 49], but more work needs to be done to better understand how pre-existing tumor resistance emerges and is maintained in different spatial structures of tumors and under different treatment strategies. Different initial distributions of resistant cell populations can cause different outcomes. Depending on the locations of fibroblasts, some cancer cells can survive under therapy. To examine how the effects of the spatial structures are governed by these factors, we developed a 2D agent-based model in which the sensitive cells were randomly distributed over the domain and the resistant cells were clumped near the center of the domain, randomly distributed over the domain, or uniformly distributed over the domain. It is reported in the literature that high time gain is associated with initial density and low resistance [22, 27]. Our results are inclined with this conclusion. In addition, our simulations showed that a clumped distribution of resistant cells forces high intra-species competition (R–R), leading to delayed tumor progression under therapy. The combination of high R–R competition and sustained R–S competition under adaptive therapy leads to an even longer time gain under adaptive therapy compared to continuous therapy.

The role of initial cell configuration in modulating the preference of adaptive therapy to continuous therapy is correlated with the initial fraction of resistance. Unlike the results of Strobl et al. [22, 27], Gallaher et al. [19] reported a significant delay in the progression under adaptive therapy even for high initial resistance. The reason for the discrepancy, in conclusion, lies in the modeling assumption of treatment strategy and the degree of resistance. In the former two studies, during the “on-treatment” period of adaptive therapy maximum tolerated dose is administered. In the latter study, a fraction of the maximum tolerated dose is administered. In the case of low resistance, Gallaher et al. [19] speculate, by the time tumor volume reduces to 50% of the initial volume, resistant cells remain sparse over the tumor domain with the low spatial competition. Alike the random and uniform cases we discussed, the tumor then grows back abruptly during the treatment holidays and relapses quicker than it does under continuous therapy. Therefore, we conclude that adaptive therapy is beneficial when the resistance is not spatially dispersed.

Our analysis of the effects of the CAF distributions suggested that fibroblasts located in the non-overlapping regions with R-cell clumps play the central role for faster progression. For resistant cells that are already competing (overlapping R-cells and CAFs), the fibroblast-mediated advantages of tumor progression are not significant. On the other hand, if fibroblasts are non-overlapping to resistant cells, resistant cells on the leading edge that experience less competition can exploit fibroblast-mediated growth, leading to much faster tumor progression in both continuous and adaptive therapy. In our simulations, fibroblasts promoted sensitive cell proliferation, which unexpectedly increased the chance of drug-induced cell death because only proliferating sensitive cells can engage in cell death. During the “off” treatment in the adaptive therapy cycles, both cell types gained the same promotion by fibroblasts. Thus, the competition between the resistant cells and sensitive cells was unexpectedly reduced, resulting in a negligible benefit of adaptive therapy compared to continuous therapy. Based on our simulations, fibroblast distribution is not a crucial factor responsible for deciding the preference of AT over CT-MTD, though it is strongly associated with faster relapse (shorter TTP).

The differential characteristics of metastatic lesions drive the evolution of tumors and the success of treatments [50–53]. A new metastatic lesion can be detected in spite of the administration of therapy. Our simulation on a virtual patient with four metastatic lesions—with one being initially undetected—predicted complex interactions between the tumor cells and fibroblasts within each metastatic lesion. Surprisingly, we demonstrated that invisible metastatic lesions can cause a rapid failure of treatments, highlighting the importance of tracking metastatic lesions during therapy. The release of a serological marker for monitoring advanced tumors, such as LDH (lactate dehydrogenase for melanoma) [54] or PSA (prostate-specific antigen for prostate cancer) [55], may be different between primary and metastatic sites or between metastatic sites [56]. Novel imaging technologies need to be developed in order to allow for frequent non-invasive monitoring of tumor burdens. Such new technologies could offer the opportunity to better understand tumors’ spatial structures.

The model presented here is an abstract representation of what might be happening in actual tumors; it focuses on spatial variations, but not how the variations arise. For example, we did not consider different microenvironmental factors, such as oxygen levels, or growth factors. The model rests on the assumption that two key tumor cell populations—drug-sensitive and drug-resistant cell populations—compete. We also assumed a uniform drug distribution, but in reality, the diffusion of a drug through a tumor tissue could result in a spatially heterogeneous drug response [7]. The adaptive strategy for the therapy used in this study considers the initial tumor volume and one threshold for stopping treatment in order to determine the on–off cycles of the treatment. However, in several studies, the maintenance and reduction of the critical volume (not necessarily the initial volume) at different levels have been reported to be beneficial [20, 21, 25]. We chose our modeling approach as a starting point in order to better understand how the spatial distributions of resistant cells and fibroblasts impact the outcomes of adaptive therapy.

In future studies, a few other dimensions, such as sequential dosing, alternating dosing, or fibroblast inhibitors, could be incorporated into adaptive treatment strategies [57]. Multidrug therapy was recently found to be promising by West and colleagues [23, 24], but they did not consider the spatial aspects of tumors. Our simulations demonstrated that fibroblasts can cause a faster failure of adaptive therapy. In tumors, fibroblasts influence the growth of the tumor cells in a spectrum of ways [58–61]. For example, in breast cancer, fibroblasts increase the growth by secreting epidermal growth factor (EGF); furthermore, the transforming growth factor-*β* (TGF-*β*) produced by the tumor cells converts fibroblasts into myofibroblasts, which increase the secretion of EGF and thus cause even more rapid tumor progression [62]. In colon cancer, TGF-*β*1 was found to promote tumor growth by helping fibroblasts to influence tumor cells [63]. Therapies designed to target fibroblasts have been proven to be successful in cases such as liver cancer [64] and prostate cancer [65]. An adaptive therapy that combines these drugs may prolong survival with lower cumulative dose rates.

## Supporting information

S1 FigS-cell dynamics under CT-MTD.(**A**) The temporal evolution of the average number of S-cell ($\bar{S}\left(t\right)$) populations under continuous therapy with initial clumped, random, and uniform cell configurations is shown in a log plot, which shows very similar growth patterns among the different cases. (**B**)The average numbers of S-cells in the VNHD of an R-cell in the 30 realizations are shown as boxplots.(TIF)

S2 FigIncreased carrying capacity reduces the benefit of adaptive therapy by reducing spatial competition.(**A**) The blue and red boxplots show the TG from the 30 couple realizations (for both AT and CT-MTD) with respect to carrying capacities of *K* = 1 and 2, respectively. The triple asterisk (***) signifies that increasing the carrying capacity significantly reduced the TG (*p* − *value* < 0.001). (**B**) The time evolution of the mean of the average number of empty sites in the VNHD of each R-cell in the 30 realizations (${\bar{N}}_{E\bar{R}}^{i}\left(t\right)$) is shown for both CT-MTD (solid lines) and AT (dashed lines); *K* = 1 (blue) and 2 (red). *K* = 2 offers a greater number of empty sites in the VNHDs of R-cells than *K* = 1.For the clumped initial cell distribution, we investigated the effect of the spatial carrying capacity on the TG. The spatial carrying capacity was characterized as *K* = 1 (each lattice point could hold one cell) or *K* = 2 (each lattice point could hold, at most, two cells, regardless of their sensitivity or resistance). When *K* = 1 was used, a total of four cells could occupy the VNHD of each cell (i.e., $N_{Sk}^{c}\left(t\right)+N_{Rk}^{c}\left(t\right)+N_{Ek}^{c}\left(t\right)=4$). For each cell in *K* = 2, a total of eight cells could occupy a VNHD, and one additional cell could be located in the respective cell’s site (i.e., $N_{Sk}^{c}\left(t\right)+N_{Rk}^{c}\left(t\right)+N_{Ek}^{c}\left(t\right)=9$). S2A Fig shows that increasing the carrying capacity significantly decreased the TG (*p* − *value* < 0.001) from a median of 139 days to a median of 7 days. Increasing the carrying capacity provided additional room for accommodation of the daughter cells, which is observed in S2B Fig. Initially, the number of empty sites in each R-cell ${\bar{N}}_{E\bar{R}}^{c}\left(t\right)$ was above 5 for *K* = 2, whereas it was below 2 for *K* = 1. Due to this ample space in their neighborhoods, R-cells hardly experienced any spatial competition and grew at a higher pace when *K* = 2 under both AT and CT-MTD. As the total cell population grew, ${\bar{N}}_{E\bar{R}}^{c}\left(t\right)$ decreased abruptly and tended to settle below 1. For *K* = 1, a similar trend was observed; however, the number of empty sites was lower than that for *K* = 2 (${\bar{N}}_{E\bar{R},K=1}^{c}\left(t\right)<{\bar{N}}_{E\bar{R},K=2}^{c}\left(t\right)$). Comparing the number of empty sites in each R-cell’s VNHD (${\bar{N}}_{E\bar{R}}^{c}\left(t\right)$) for AT in the case of *K* = 1 with that in the case of *K* = 2 (S2B Fig, dotted lines)), we observed that, for *K* = 1, ${\bar{N}}_{E\bar{R}}^{c}\left(t\right)$ went through ups and downs several times, which suggested spatial competition with neighboring cells. On the other hand, for *K* = 2, this value monotonically decreased, and there was a very slight difference due to AT and CT-MTD. Therefore, we concluded that the short TG with *K* = 2 was due to the lack of spatial competition. We observed that the probabilities of having a negative TG were 0.03 and 0.4 for *K* = 1 and 2, respectively, i.e., an increase in carrying capacity reduces the benefit of AT over CT-MTD.(TIF)

S3 Fig Area of impact. The gray square shows the region 10 sites inside from the boundary. The orange square depicts the initial location of the R-cell clump. The pink lines (both solid and dashed) show the fibroblast region. Fibroblast regions bounded by the solid pink lines shows the area of impact.
(TIF)

S4 FigFibroblast mediated growth for *α* = 4 with clumped initial R-cell distribution.(**A**) The time evolution of the average of the total cell population ($\bar{N}\left(t\right)$) under CT-MTD in the 30 simulations is shown for all types of fibroblast configurations. (**B**) Boxplot of the TTP (time to progression) under CT-MTD in the 30 realizations. (**C**) Boxplot of the time gain under AT in the 30 realizations. (**D**) Correlation of TTP with the area of impact is shown. The circles and the asterisks (the colors are similar to the legend in A) show the TTP under CT-MTD and AT respectively. And the solid and dashed lines are the respective regression lines. The *p* − *values* for AT and CT-MTD indicate significance of the correlation. (**E**) Time gain (TG) is shown as boxplots for all type of fibroblast structures.(TIF)

S5 Fig Complex dynamics of multiple metastases under AT and CT-MTD. The time evolution of the total cell population in the four metastases is shown in the sub-figures. The first, second, and third rows show the results for Cases II, III, and IV, respectively. The first and second columns show the results for clumped and random initial cell configurations in the invisible metastasis, respectively. In each sub-figure, the blue, red, yellow, and black colors show the total cell populations in metastasis 1, metastasis 2, metastasis 3, and metastasis 4, respectively; the vertical cyan lines show the emergence time (ET) of the invisible metastasis, and the red line shows the TTP. The solid and dashed lines show the results under CT-MTD and AT, respectively.
(TIF)

S1 Movie Temporal change of tumor configurations for different initial R-cell configurations. The video shows the evolution of tumor for clumped (first column), random (second column), and uniform (third column) initial cell configurations with time under CT-MTD (first row) and AT (second row). The red, blue, and white dots correspond to R-cells, S-cells, and empty sites respectively.
(MP4)

S2 Movie Temporal change of cell configuration for different fibroblast distributions. The video shows the evolution of tumor for different fibroblast structures (column wise) for clumped initial cell configuration under CT-MTD (first row) and AT (second row). The red, blue, and white dots correspond to R-cells, S-cells, and empty sites respectively.
(MP4)

S3 Movie Temporal change of cell configuration of different metastatic lesions for case I (Fig 11) with clumped invisible metastasis. The red, blue, and white dots correspond to R-cells, S-cells, and empty sites respectively. The gray dots show the sites that are accompanied by CAFs. The first and second row show results for CT-MTD and AT respectively.
(MP4)

S4 Movie Temporal change of cell configurations of different metastatic lesions for case I (Fig 11) with random invisible metastasis. The red, blue, and white dots correspond to R-cells, S-cells, and empty sites respectively. The gray dots show the sites that are accompanied by CAFs. The first and second row show results for CT-MTD and AT respectively.
(MP4)
