# Supplementary figures and images for: The impact of the spatial heterogeneity of resistant cells and fibroblasts on treatment response
Source: PLoS Comput Biol. 2022 Mar 9;18(3):e1009919. doi: 10.1371/journal.pcbi.1009919 (PMC8906648; doi:10.1371/journal.pcbi.1009919)

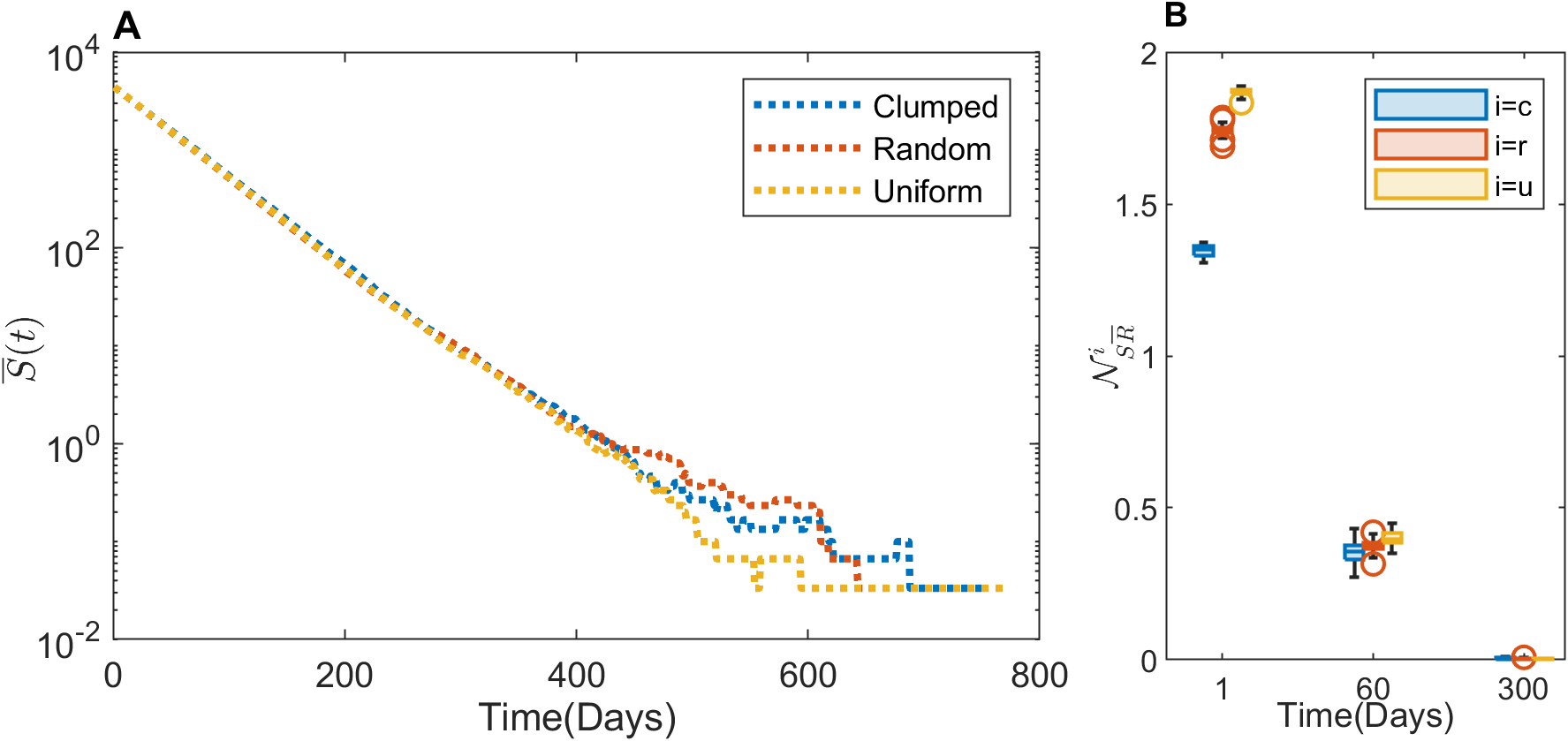

Supplement: S1 Fig — (A) The temporal evolution of the average number of S-cell (S¯(t)) populations under continuous therapy with initial clumped, random, and uniform cell configurations is shown in a log plot, which shows very similar growth patterns among the different cases. (B)The average numbers of S-cells in the VNHD of an R-cell in the 30 realizations are shown as boxplots. (TIF) [file pcbi.1009919.s001.tif]

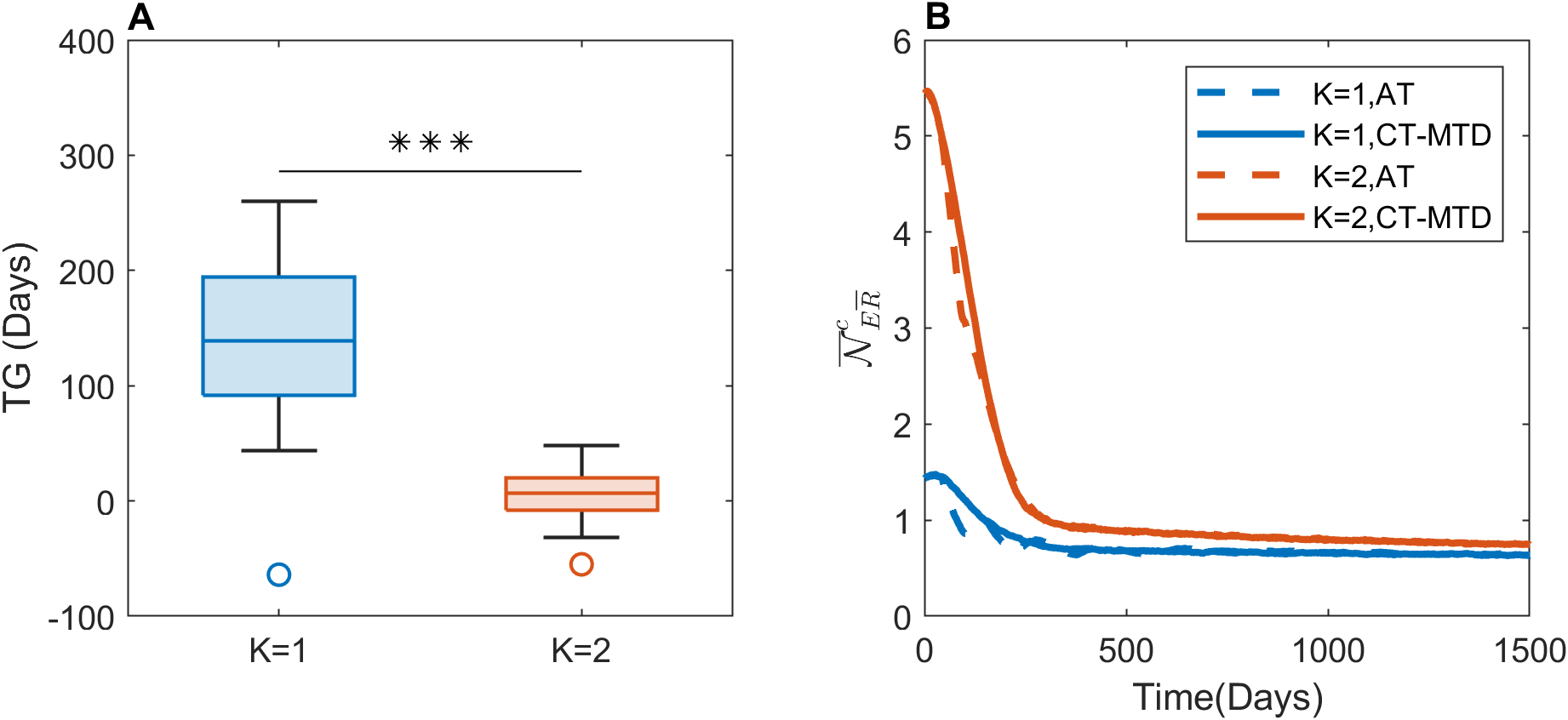

Supplement: S2 Fig — (A) The blue and red boxplots show the TG from the 30 couple realizations (for both AT and CT-MTD) with respect to carrying capacities of K = 1 and 2, respectively. The triple asterisk (***) signifies that increasing the carrying capacity significantly reduced the TG (p − value < 0.001). (B) The time evolution of the mean of the average number of empty sites in the VNHD of each R-cell in the 30 realizations (N¯ER¯i(t)) is shown for both CT-MTD (solid lines) and AT (dashed lines); K = 1 (blue) and 2 (red). K = 2 offers a greater number of empty sites in the VNHDs of R-cells than K = 1. For the clumped initial cell distribution, we investigated the effect of the spatial carrying capacity on the TG. The spatial carrying capacity was characterized as K = 1 (each lattice point could hold one cell) or K = 2 (each lattice point could hold, at most, two cells, regardless of their sensitivity or resistance). When K = 1 was used, a total of four cells could occupy the VNHD of each cell (i.e., NSkc(t)+NRkc(t)+NEkc(t)=4). For each cell in K = 2, a total of eight cells could occupy a VNHD, and one additional cell could be located in the respective cell’s site (i.e., NSkc(t)+NRkc(t)+NEkc(t)=9). S2A Fig shows that increasing the carrying capacity significantly decreased the TG (p − value < 0.001) from a median of 139 days to a median of 7 days. Increasing the carrying capacity provided additional room for accommodation of the daughter cells, which is observed in S2B Fig. Initially, the number of empty sites in each R-cell N¯ER¯c(t) was above 5 for K = 2, whereas it was below 2 for K = 1. Due to this ample space in their neighborhoods, R-cells hardly experienced any spatial competition and grew at a higher pace when K = 2 under both AT and CT-MTD. As the total cell population grew, N¯ER¯c(t) decreased abruptly and tended to settle below 1. For K = 1, a similar trend was observed; however, the number of empty sites was lower than that for K = 2 (N¯ER¯,K=1c(t)<N¯ER¯,K=2c(t)). Compari [file pcbi.1009919.s002.tif]

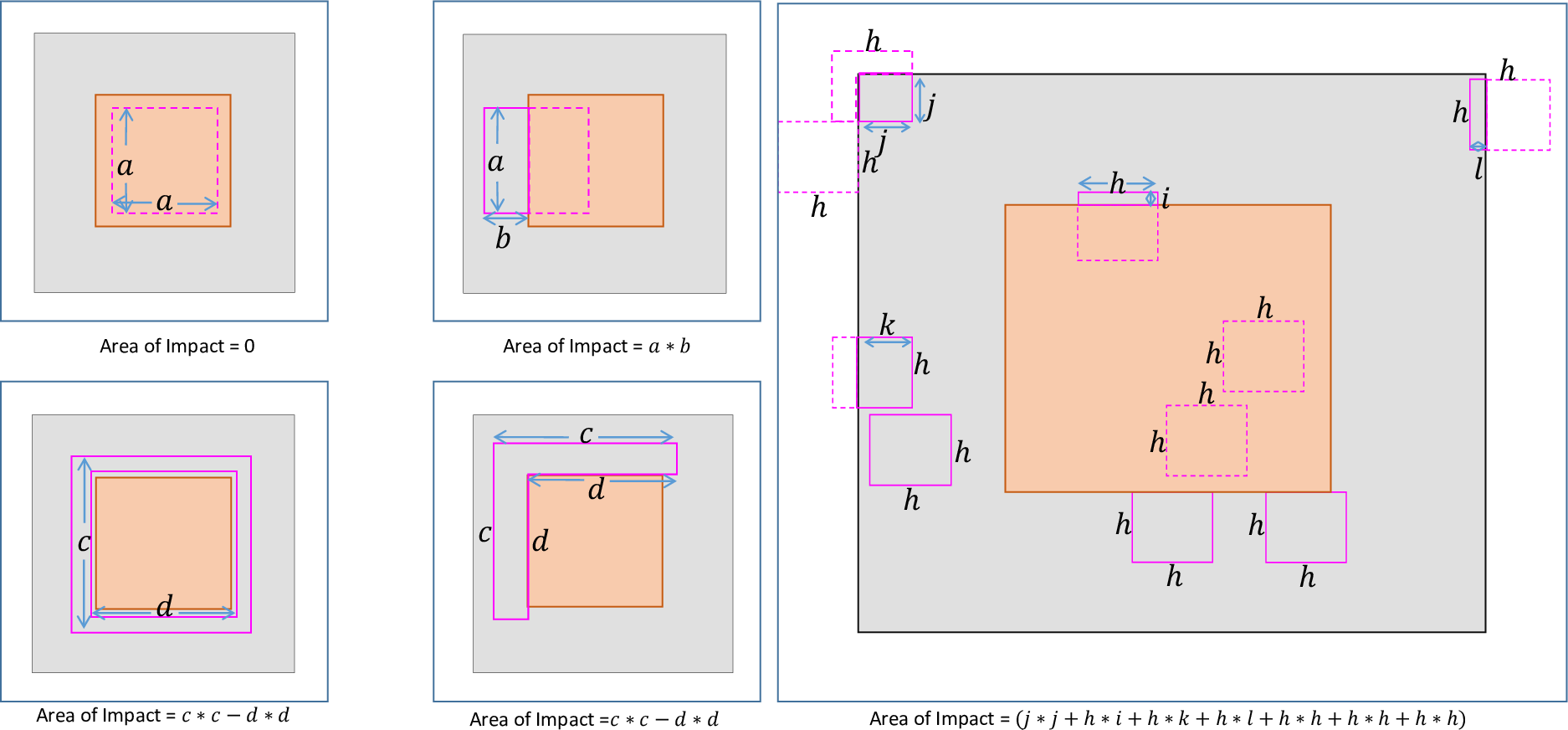

Supplement: S3 Fig — The gray square shows the region 10 sites inside from the boundary. The orange square depicts the initial location of the R-cell clump. The pink lines (both solid and dashed) show the fibroblast region. Fibroblast regions bounded by the solid pink lines shows the area of impact. (TIF) [file pcbi.1009919.s003.tif]

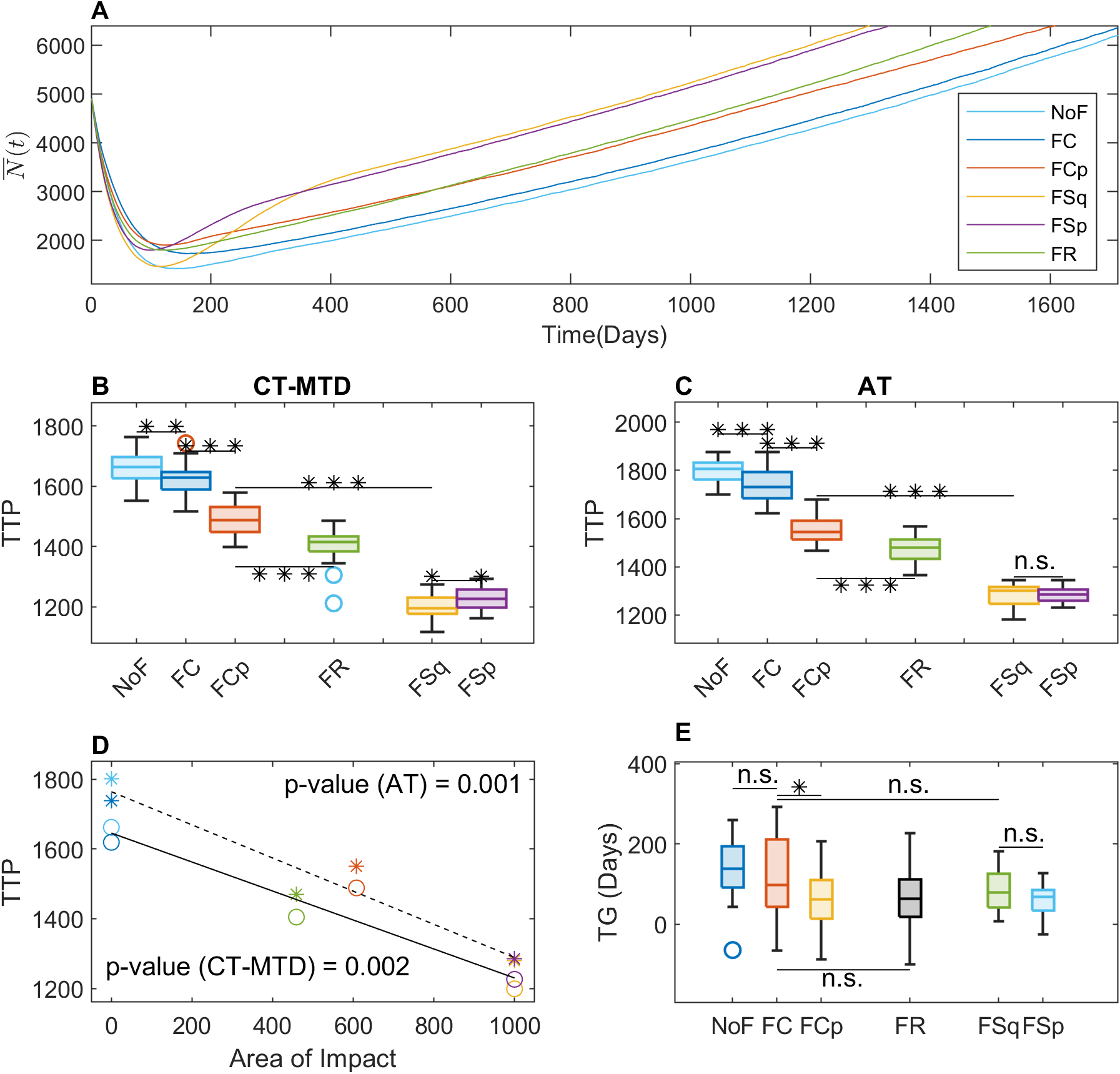

Supplement: S4 Fig — (A) The time evolution of the average of the total cell population (N¯(t)) under CT-MTD in the 30 simulations is shown for all types of fibroblast configurations. (B) Boxplot of the TTP (time to progression) under CT-MTD in the 30 realizations. (C) Boxplot of the time gain under AT in the 30 realizations. (D) Correlation of TTP with the area of impact is shown. The circles and the asterisks (the colors are similar to the legend in A) show the TTP under CT-MTD and AT respectively. And the solid and dashed lines are the respective regression lines. The p − values for AT and CT-MTD indicate significance of the correlation. (E) Time gain (TG) is shown as boxplots for all type of fibroblast structures. (TIF) [file pcbi.1009919.s004.tif]

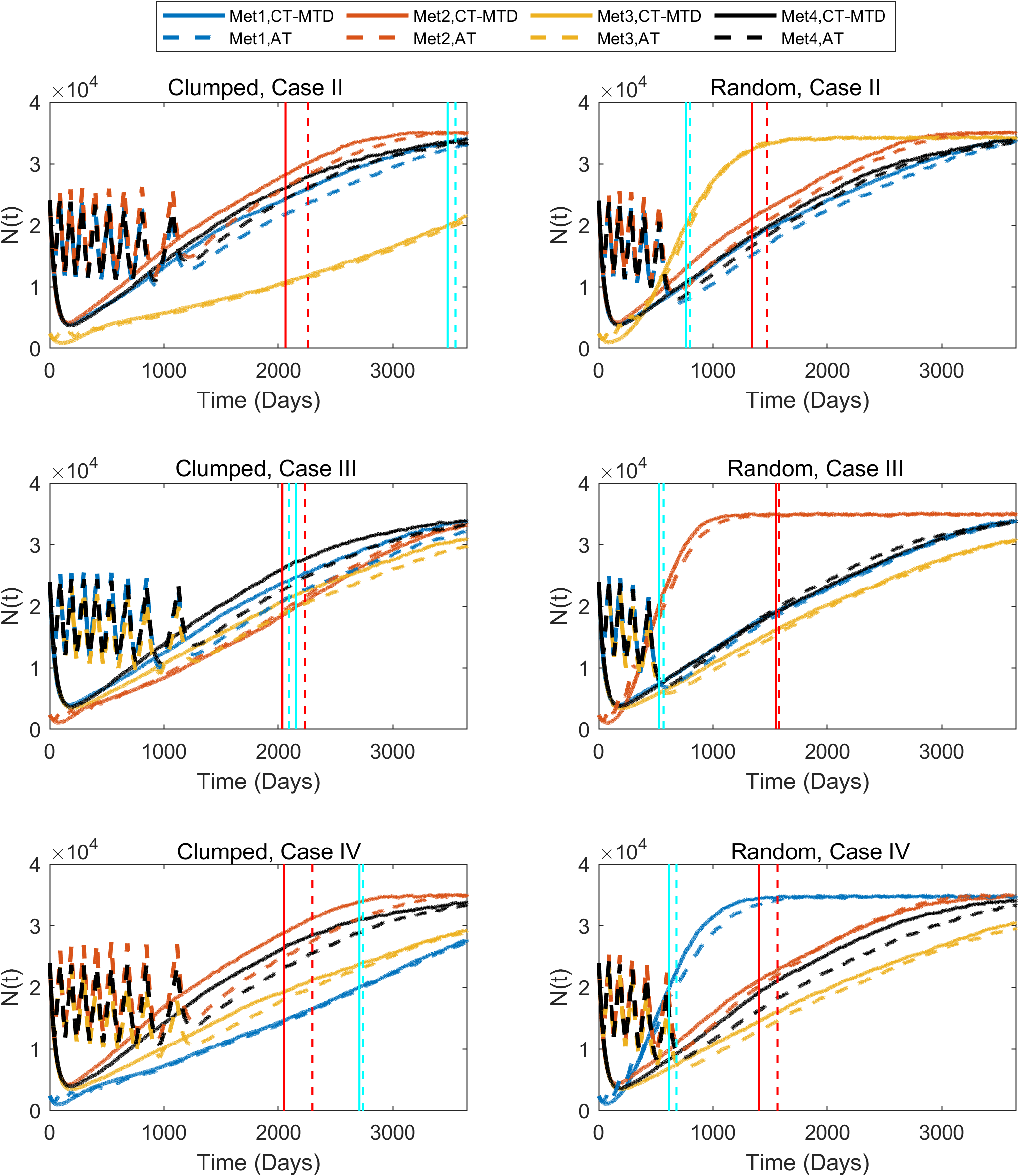

Supplement: S5 Fig — The time evolution of the total cell population in the four metastases is shown in the sub-figures. The first, second, and third rows show the results for Cases II, III, and IV, respectively. The first and second columns show the results for clumped and random initial cell configurations in the invisible metastasis, respectively. In each sub-figure, the blue, red, yellow, and black colors show the total cell populations in metastasis 1, metastasis 2, metastasis 3, and metastasis 4, respectively; the vertical cyan lines show the emergence time (ET) of the invisible metastasis, and the red line shows the TTP. The solid and dashed lines show the results under CT-MTD and AT, respectively. (TIF) [file pcbi.1009919.s005.tif]
